# Multistep conformational changes leading to the gate opening of light-driven sodium pump rhodopsin

**DOI:** 10.1016/j.jbc.2023.105393

**Published:** 2023-10-27

**Authors:** Yukino Sato, Tsubasa Hashimoto, Koji Kato, Akiko Okamura, Kaito Hasegawa, Tsukasa Shinone, Yoshikazu Tanaka, Yoshiki Tanaka, Tomoya Tsukazaki, Takashi Tsukamoto, Makoto Demura, Min Yao, Takashi Kikukawa

**Affiliations:** 1 Graduate School of Life Science, Hokkaido University, Sapporo, Japan; 2 Research Institute for Interdisciplinary Science, Okayama University, Okayama, Japan; 3 Graduate School of Life Sciences, Tohoku University, Sendai, Japan; 4 Graduate School of Biological Sciences, Nara Institute of Science and Technology, Nara, Japan; 5 Faculty of Advanced Life Science, Hokkaido University, Sapporo, Japan

**Keywords:** membrane transport, sodium transport, sodium pump, photobiology, rhodopsin, ion pump, retinal proteins

## Abstract

Membrane transport proteins require a gating mechanism that opens and closes the substrate transport pathway to carry out unidirectional transport. The "gating" involves large conformational changes and is achieved *via* multistep reactions. However, these elementary steps have not been clarified for most transporters due to the difficulty of detecting the individual steps. Here, we propose these steps for the gate opening of the bacterial Na^+^ pump rhodopsin, which outwardly pumps Na^+^ upon illumination. We herein solved an asymmetric dimer structure of Na^+^ pump rhodopsin from the bacterium *Indibacter alkaliphilus*. In one protomer, the Arg108 sidechain is oriented toward the protein center and appears to block a Na^+^ release pathway to the extracellular (EC) medium. In the other protomer, however, this sidechain swings to the EC side and then opens the release pathway. Assuming that the latter protomer mimics the Na^+^-releasing intermediate, we examined the mechanism for the swing motion of the Arg108 sidechain. On the EC surface of the first protomer, there is a characteristic cluster consisting of Glu10, Glu159, and Arg242 residues connecting three helices. In contrast, this cluster is disrupted in the second protomer. Our experimental results suggested that this disruption is a key process. The cluster disruption induces the outward movement of the Glu159-Arg242 pair and simultaneously rotates the seventh transmembrane helix. This rotation resultantly opens a space for the swing motion of the Arg108 sidechain. Thus, cluster disruption might occur during the photoreaction and then trigger sequential conformation changes leading to the gate-open state.

Membrane transport proteins fulfill vital roles by exchanging various materials in and out of the cells. One-way transport of the substrate requires gating machinery, which controls the accessibility of the substrate-binding site to the medium. As proposed in the alternative access model (1), this accessibility should be switched alternatively from one side of the membrane to the other. For substrate uptake, the "entrance" gate is opened to expose the binding site to the medium. After substrate uptake, "accessibility switching" occurs for substrate release. In this step, the entrance gate is first closed, and then the other "exit" gate is opened to expose the binding site to the opposite medium. Thus, the gating machinery plays essential roles in accessibility switching. The "gating" should involve large-scale structural alterations and thus require multiple steps of conformational changes. However, these steps are not clarified for most transport proteins, reflecting the difficulty in detecting stepwise conformational changes. Here, we report the plausible steps leading to "exit" gate opening for the light-driven Na^+^ pump rhodopsin (NaR).

NaR belongs to the microbial rhodopsin family, whose members are widespread in the microbial world (2, 3, 4). Similar to animal rhodopsins, microbial rhodopsins consist of seven transmembrane helices and the chromophore retinal to acquire light energy. Animal rhodopsins mainly act as light sensors. In contrast, microbial rhodopsins are multifunctional: they function not only as light sensors but also as light-driven ion pumps, light-gated ion channels, and even light-switchable enzymes (5, 6, 7). In the dark state, all microbial rhodopsins contain all-*trans* retinal, which is bound to a specific Lys residue on the seventh helix (G helix) *via* a protonated Schiff base (PSB). Upon light absorption, the retinal isomerizes to the 13-*cis* configuration, which distorts the protein structure. This energized state of protein thermally relaxes to the original dark state through several structural intermediates. During this cyclic photoreaction called the photocycle, microbial rhodopsins fulfill their respective functions. Figure 1*A* shows a typical photocycle scheme of NaR (2, 8, 9). During this photocycle, NaR achieves outward Na^+^ transport. NaR is widely spread in marine bacteria and thought to maintain the smaller Na^+^ concentration in the cytoplasm (CP) and/or drive secondary transporters for nutrition imports.

**Figure 1:**
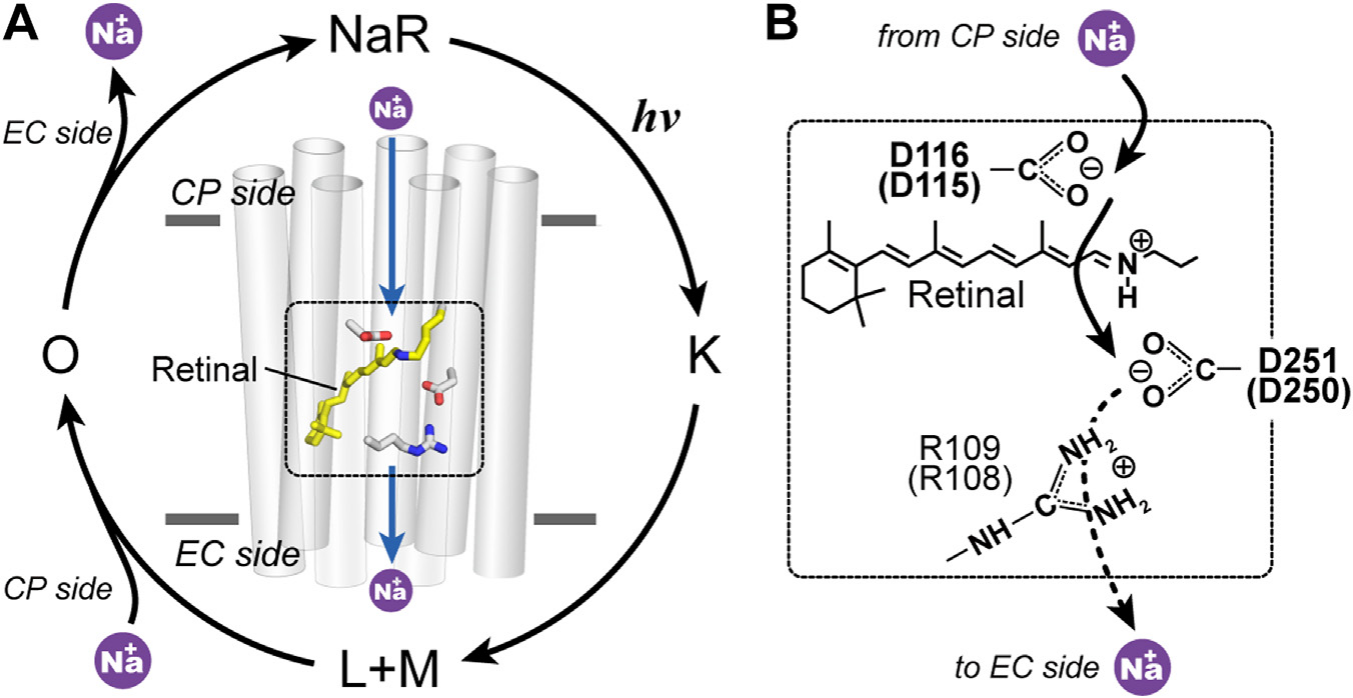
**Overview of the Na**^**+**^**translocation of NaR.***A*, outline of the photocycle. Only the major intermediates are shown, and "L+M" indicates their quasi-equilibrium. Na^+^ is captured and released during the formation and decay of the O intermediate. *B*, schematic of the positions of two Asp residues composing putative Na^+^-binding sites at the O intermediate. The Na^+^ release pathway after the second binding site appears to be blocked by the Arg residue. The residue numbers are those of KR2. In parentheses are the corresponding numbers of IaNaR. These residues are also schematically shown in (*A*). CP, cytoplasmic; EC, extracellular; IaNaR, *Inidibacter alkaliphilus* sodium pump rhodopsin; KR2, *Krokinobacter eikastus* rhodopsin 2; NaR, sodium pump rhodopsin.

NaR is the third identified ion-pumping rhodopsin and has unique differences from the other H^+^ and Cl^-^ pumps (3, 5). In the dark state, these two ion pumps already bind the substrate ions in the vicinities of the PSBs, that is, the reaction center of rhodopsin. In contrast, NaR does not bind the substrate Na^+^ in the dark state (2). After starting the photocycle, NaR captures Na^+^ from CP medium and then releases it to the extracellular (EC) medium (Fig. 1*A*). Thus, in the unphotolyzed state, its Na^+^-binding site is not accessible to both the CP and EC sides.

Photolyzed NaR captures and releases Na^+^ during the formation and decay of the O intermediate (Fig. 1*A*) (10). Thus, during these steps, entrance and exit gates should control the accessibility of the Na^+^-binding site to the medium. Currently, two Na^+^-binding sites have been proposed based on crystallographic studies by two research groups (11, 12). They determined the intermediate structures of NaR from the bacterium *Krokinobacter eikastus* (hereafter, this NaR is abbreviated as KR2). Both structures involved Na^+^ and were thus assigned to the O intermediate. However, the binding sites were different: each Na^+^ was bound to a different Asp residue. The positions of two Asp residues are schematically indicated in Figure 1*B*. The detailed structures are shown in Fig. S1, *B* and *C*, which also involves the dark state structure (Fig. S1*A*) and the structure of the last intermediate (Fig. S1*D*). In one O-intermediate (Fig. S1*B*), which was trapped by the cryo-trapping method, Na^+^ binds in the vicinity of the Asp116 residue (Fig. 1*B*) (11). In the other O intermediate (Fig. S1*C*), whose structure was solved by time-resolved crystallography, Na^+^ binds near the Asp251 residue on the EC side of the PSB (Fig. 1*B*) (12). The relationship between these two structures has not been clarified. However, given the location of these sites, Na^+^ is probably transported through both binding sites. This view is consistent with our previous photochemical analyses showing that the O intermediate is divided into two substates, O1 and O2 (9, 13, 14).

In the dark state, PSB strongly interacts with the Asp116 residue and seems to block the approach of Na^+^ from the CP side (Fig. S1*A*). The photoinduced isomerization of the retinal enforces the displacement of PSB, which probably allows the Na^+^ approach. Thus, the PSB itself might act as the main component of the entrance gate. In contrast, for the exit gate, the Arg109 residue might play an essential role. As shown in Figure 1*B* and Fig. S1*C*, this residue is located just below the second Na^+^-binding site and seems to block the Na^+^ release pathway. This residue still orients toward the binding site even in the O intermediate structure (Fig. S1, *B* and *C*) (11, 12). However, in the last intermediate (Fig. S1*D*), this residue swings toward the EC side, and simultaneously, Na^+^ disappears (12). Thus, for Na^+^ release, the swing motion of the Arg sidechain seems to be necessary, and this residue probably acts as the gate itself.

In this study, we propose a model for the multistep conformational changes leading to the swing motion of the Arg sidechain, that is, gate opening for Na^+^ release. KR2 was the only NaR whose tertiary structure was reported. We herein solved the structure of a NaR from the bacterium *Indibacter alkaliphilus* (hereafter, this NaR is abbreviated as IaNaR) and then found unique features in one protomer of the asymmetric dimer. In that protomer, the Arg108 residue (corresponding to Arg109 of KR2) orients toward the EC side. Thus, the Na^+^-binding site near Asp250 (corresponding to Asp251 of KR2) is open to the EC medium. This is in clear contrast to the other protomer, in which the Arg108 sidechain orients toward the binding site and thus blocks the Na^+^ release pathway, similar to the unphotolyzed KR2 (Figs. 1*B* and S1*A*). Thus, assuming that this "unique" structure mimics the Na^+^-releasing intermediate, we examined plausible steps of conformational changes leading to the "open" structure. The results indicate that a structural change at the EC surface triggers sequential conformation changes that eventually lead to the gate-open structure.

## Results

### Overall structure of IaNaR

**Figure 2:**
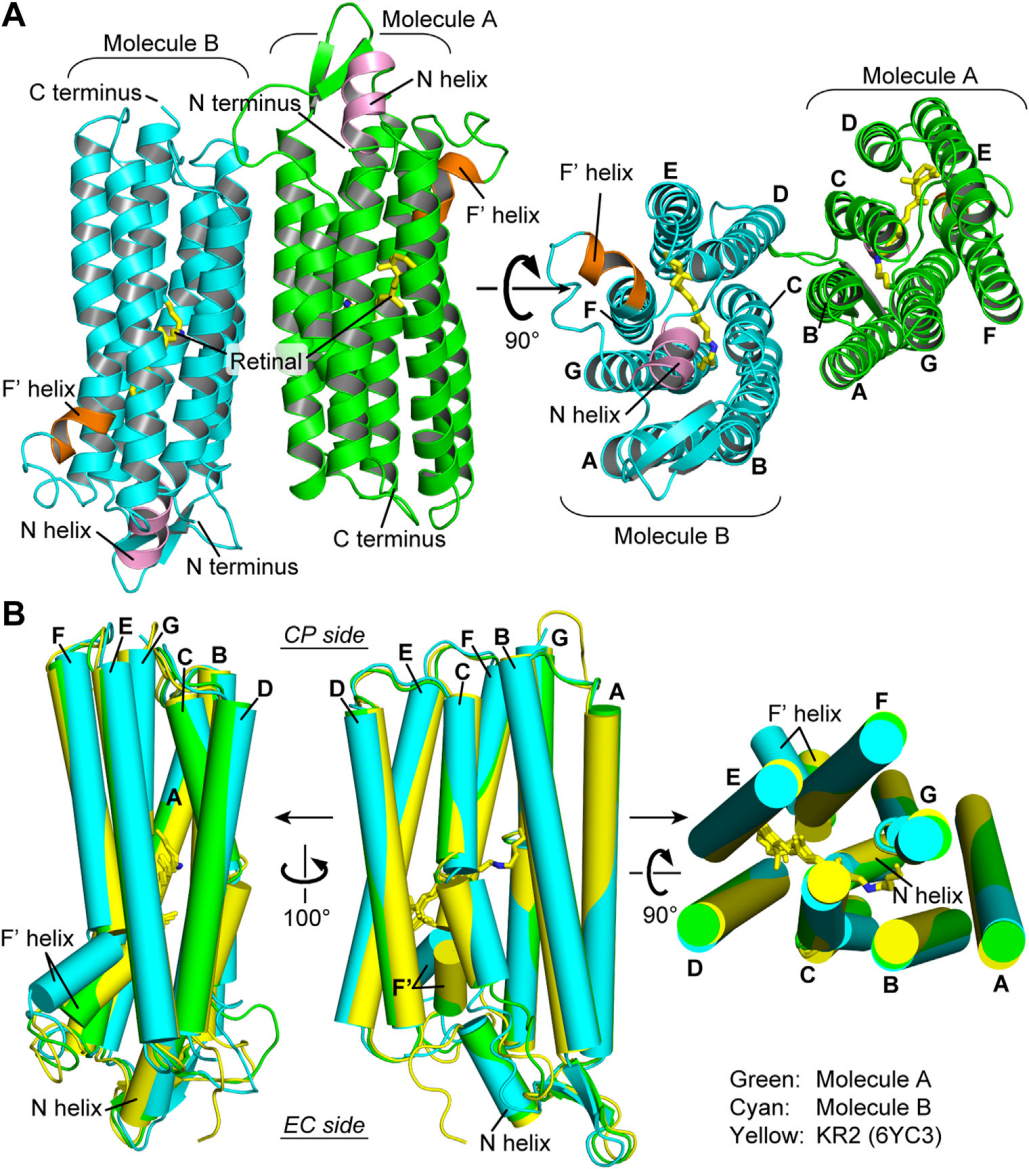
**Overall structure of IaNaR and comparison with KR2.***A*, crystal structure of the antiparallel IaNaR dimer viewed parallel to the membrane (*left*) and normal to the membrane surface (*right*). Seven transmembrane helices are labeled with A–G, and the "N helix" and "F' helix" are colored in *pink* and *orange*, respectively. The all-*trans* retinals, depicted by stick models, are colored in *yellow*. *B*, superimposed transmembrane helices of two IaNaR protomers (*green* and *cyan*) and KR2 (*yellow*), viewed parallel to the membrane from two angles (*left* and *middle*) and viewed from the EC side (*right*). The F′ helix of IaNaR molecule B (*cyan*) is largely bent outward. CP, cytoplasmic; EC, extracellular; IaNaR, *Inidibacter alkaliphilus* sodium pump rhodopsin; KR2, *Krokinobacter eikastus* rhodopsin 2.

Various aspects of NaR have been clarified through studies on KR2. To further understand the mechanism of NaR, we herein determined the crystal structure of IaNaR. The crystals were obtained by the lipidic cubic phase (LCP) at pH 5.1 (15). The structure was determined by the molecular replacement method using the coordinates of KR2 (PDB ID: 3X3C) (16) and refined to 2.8 Å resolution (Table S1). The crystals belonged to the *P*2_1_2_1_2_1_ space group, and the asymmetric unit contained two antiparallel protomers (Fig. 2*A*), which will be referred to as molecules A and B, respectively. Except for the several disordered amino- and carboxy-terminal residues, other residues (molecule A, residues 8–269; molecule B, residues 6–270) are clearly visualized in the electron density map together with all-*trans* retinal and several water molecules. The overall structures of the two protomers were similar to the reported KR2 structures (Fig. 2*B*), that is, they commonly contain a short N-terminal helix (N helix; residues 9–17) and consist of typical seven transmembrane domains. Moreover, the EC ends of the F helices (F′ helices) commonly bend outward.

**Figure 3:**
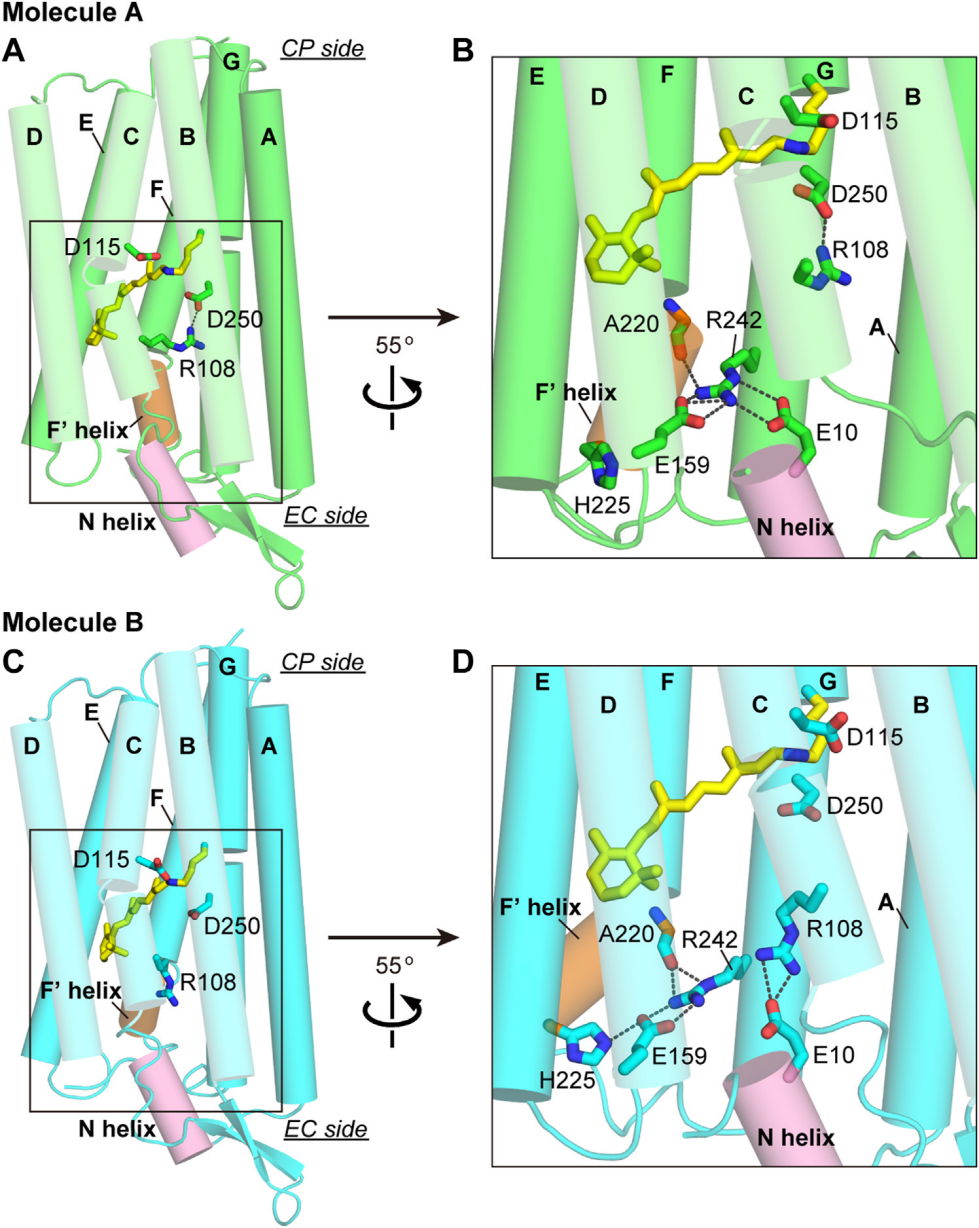
**Comparison of the structures of two IaNaR protomers.** The overall structures of molecules A and B are shown on the *left* (*A* and *C*), and their boxed regions are rotated 55° and enlarged on the *right* (*B* and *D*). In molecule A, the Arg108 sidechain is oriented *upward*, and the three-residue cluster (Glu10, Glu159 and Arg242) connects the N helix, *D* and *G* transmembrane helices at the EC surface. In molecule B, the Arg108 sidechain is oriented downward, and the cluster is disrupted. Corresponding figures for KR2 are shown in Fig. S2. CP, cytoplasmic; EC, extracellular; IaNaR, *Inidibacter alkaliphilus* sodium pump rhodopsin; KR2, *Krokinobacter eikastus* rhodopsin 2.

Out of the two protomers, molecule A indeed has a close structure with KR2 even at a more detailed level. However, molecule B has distinct features from them, especially at the EC half side. Figure 3 compares these parts between molecules A and B. The corresponding parts of KR2 are shown in Fig. S2. Similar to KR2 (Fig. S2), the F' helix of molecule A (Fig. 3, *A* and *B*) is outwardly bent slightly, and the Arg108 sidechain (corresponding to Arg109 of KR2) points upward. Meanwhile, at the EC side surface of molecule A, there exists a characteristic cluster consisting of Glu10, Glu159, and Arg242 residues (Fig. 3*B*). As a result, three helices, that is, the N helix and D and G transmembrane helices, are connected to each other. In KR2 (Fig. S2*B*), the same cluster consisted of Glu11, Glu160, and Arg243 residues. In contrast, in molecule B (Fig. 3, *C* and *D*), the F' helix largely bends outward, which disrupts the cluster of three residues at the EC surface. Moreover, the Arg108 sidechain points downward, which is opposite to the direction of Arg108 in molecule A. These differences inspired us to perform further experiments, where we assumed that molecule B mimics the Na^+^-releasing intermediate. Those details will be described after comparing other parts among two IaNaR protomers and KR2.

### Comparison with KR2 structures

**Figure 4:**
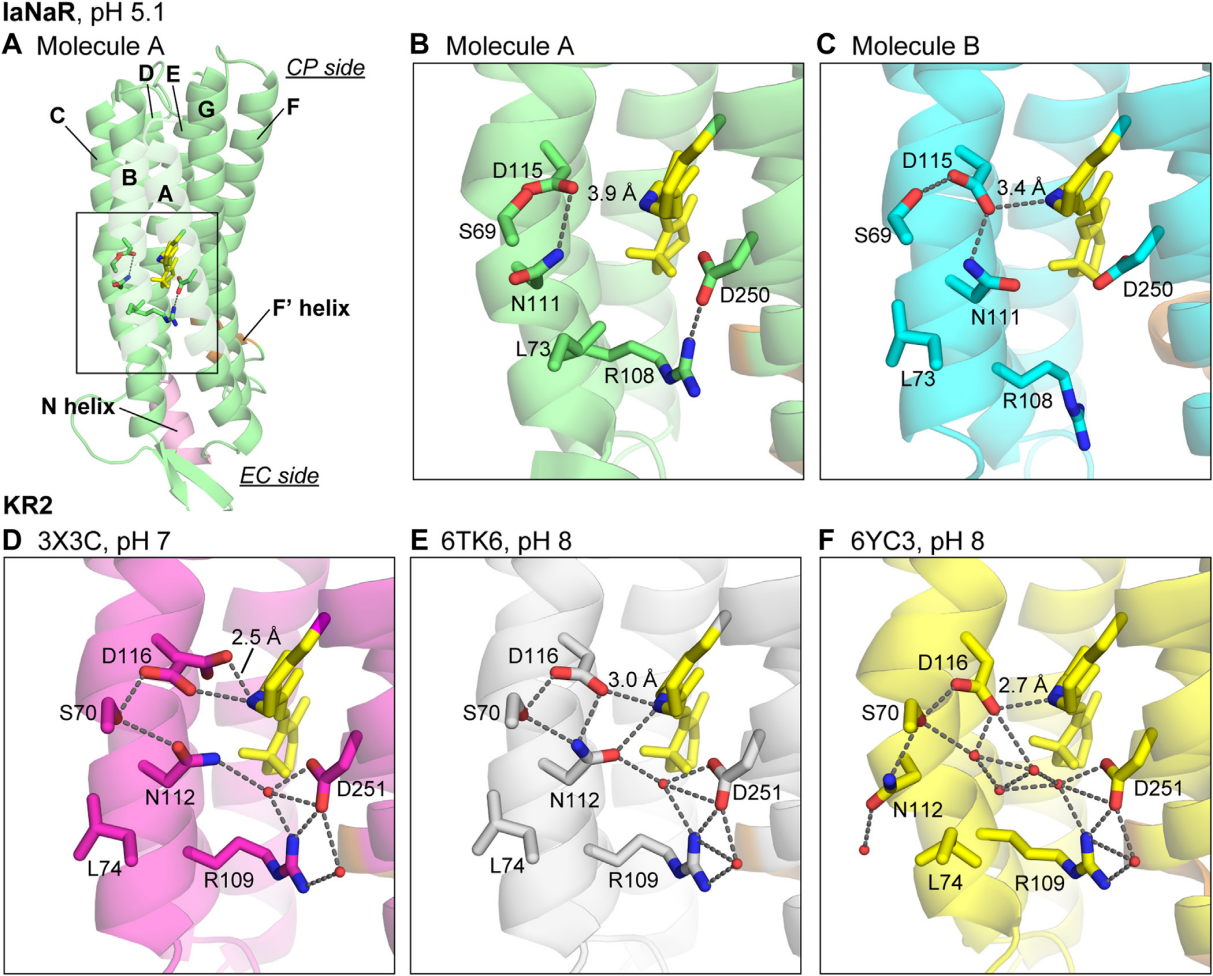
**Structural comparison of the retinal Schiff base regions.***A*, the overall structure of IaNaR molecule A. Its boxed region is enlarged in (*B*). The corresponding figure for molecule B is shown in (*C*). The lower panels (*D*–*F*) also show the corresponding regions of three representative KR2 structures. Their PDB codes are indicated above the panels. The hydrogen bonds are indicated with broken *lines*. Helices A and B are not shown in the enlarged panels (*B*–*F*). CP, cytoplasmic; EC, extracellular; IaNaR, *Inidibacter alkaliphilus* sodium pump rhodopsin; KR2, *Krokinobacter eikastus* rhodopsin 2.

The other moieties of molecules A and B have similar structures to KR2. In particular, concerning the CP half sides of these molecules (Fig. S3), there are no essential differences in the positions of Cα atoms and the orientations of functionally important residues, whose representatives are also shown in Fig. S3 (8, 17). Meanwhile, there are some differences in the vicinity of the PSB (Fig. 4). In this region, there are several variations even among the KR2 structures reported thus far. These regions of molecules A and B are shown in Figure 4, *B* and *C*, and those of the representative KR2 structures (11, 12, 16) are shown in the lower panels. In Figure 4, *D* and *E*, KR2 is in the monomeric state, whereas it is in the pentameric state in Figure 4*F*. The characteristic differences among these three KR2 structures are the orientations of the Asp116 and Asn112 sidechains. For Asp116, two different orientations are observed in Figure 4*D*, reflecting that this crystal was first obtained at pH 4 and then soaked in the pH 7 solution (16). Of the two orientations, the downward orientation was also observed in the structure at pH 4. Thus, the other one reflects the orientation at pH 7. In contrast, in other KR2 structures at pH 8 (Fig. 4, *E* and *F*), only downward orientations are observed while maintaining close distances between the carboxyl oxygen of Asp116 and the Schiff base nitrogen. These distances in the three KR2 structures are 2.5 to 3.0 Å (Fig. 4, *D*–*F*). For IaNaR (Fig. 4, *B* and *C*), the corresponding Asp115 is also oriented downward in both protomers, while the distances to the Schiff base nitrogen are somewhat longer (3.9 Å and 3.4 Å for molecules A and B, respectively). Thus, in both protomers, Asp115 has a weaker interaction with PSB. The long distances might be caused by crystallization at pH 5.1. As mentioned below, IaNaR exhibits a spectral redshift by lowering the pH from 9 to 5. This shift reflects the protonation of Asp115 and is accompanied by a color change from pink to purple. Our crystal also had a purple color. Thus, in this crystal, the Asp115 residue is probably protonated, and the resultant weaker interaction with PSB might elongate their distance. We soaked our crystal in pH 7 to 10 buffer solutions and then performed crystallographic analyses. However, the crystal did not diffract, so the structures could not be determined. Thus, further discussion seems to be difficult. If we obtain fine crystals at neutral or alkaline pH, a closer distance between Asp115 and PSB might appear, as observed in KR2.

Asn112 is the other residue whose sidechain orientation is characteristically different among the KR2 structures (Fig. 4, *D*–*F*). For the structures in the monomeric state (Fig. 4, *D* and *E*), the Asn112 sidechain orients toward the PSB and forms hydrogen bonding interactions with the PSB or the water molecule. In contrast, in the KR2 structure in the pentameric form (Fig. 4*F*), the Asn112 sidechain orients outside of the protein, and the resulting opened space fills several water molecules. This structure is called the "expanded form" and has been observed only when KR2 is crystallized in the pentameric form at neutral or alkaline pH (18, 19). KR2 is considered to be in the pentameric form in the native environment (20). Thus, the expanded form might be closer to the natural dark state than the "compact form" in the other two KR2 structures (Fig. 4, *D* and *E*), although the functional consequences of these forms are not fully clarified. For the IaNaR structure (Fig. 4, *B* and *C*), the corresponding Asn111 sidechain has different orientations in the two protomers. In molecule B (Fig. 4*C*), its sidechain orients toward PSB. Thus, this configuration can be categorized into the compact form (Fig. 4, *D* and *E*). In contrast, Asn111 in molecule A (Fig. 4*B*) has a somewhat similar orientation to that in the expanded form (Fig. 4*F*), although water molecules are not observed in the opened space. Thus, the pentameric state might not be mandatory for the expanded form. For further discussion, additional information such as the IaNaR structure at neutral or alkaline pH seems to be necessary.

### Working hypothesis of the "exit" gate-opening mechanism for Na^+^ release

As mentioned above, in molecule B (Fig. 3*D*), the three-residue cluster at the EC surface is disrupted, and in the vicinity of the PSB, the Arg108 sidechain lacks the interaction with Asp250 and is oriented toward the EC side. These differences from molecule A seem to be related to the large outward bending of the F' helix caused by unnatural interprotomer interactions. As shown in Fig. S4, the EC end of this F' helix forms a hydrophobic interaction with the A helix (the first helix) from molecule B of the neighboring asymmetric unit. Thus, the unique structure of molecule B (Fig. 3*D*) does not reflect the natural dark state. However, the downward movement of the Arg108 sidechain seems to be necessary for Na^+^ release, as mentioned above. Thus, we assumed that molecule B mimics the Na^+^-releasing intermediate and then proposed a working hypothesis for the mechanism leading to the swing motion of the Arg sidechain.

**Figure 5:**
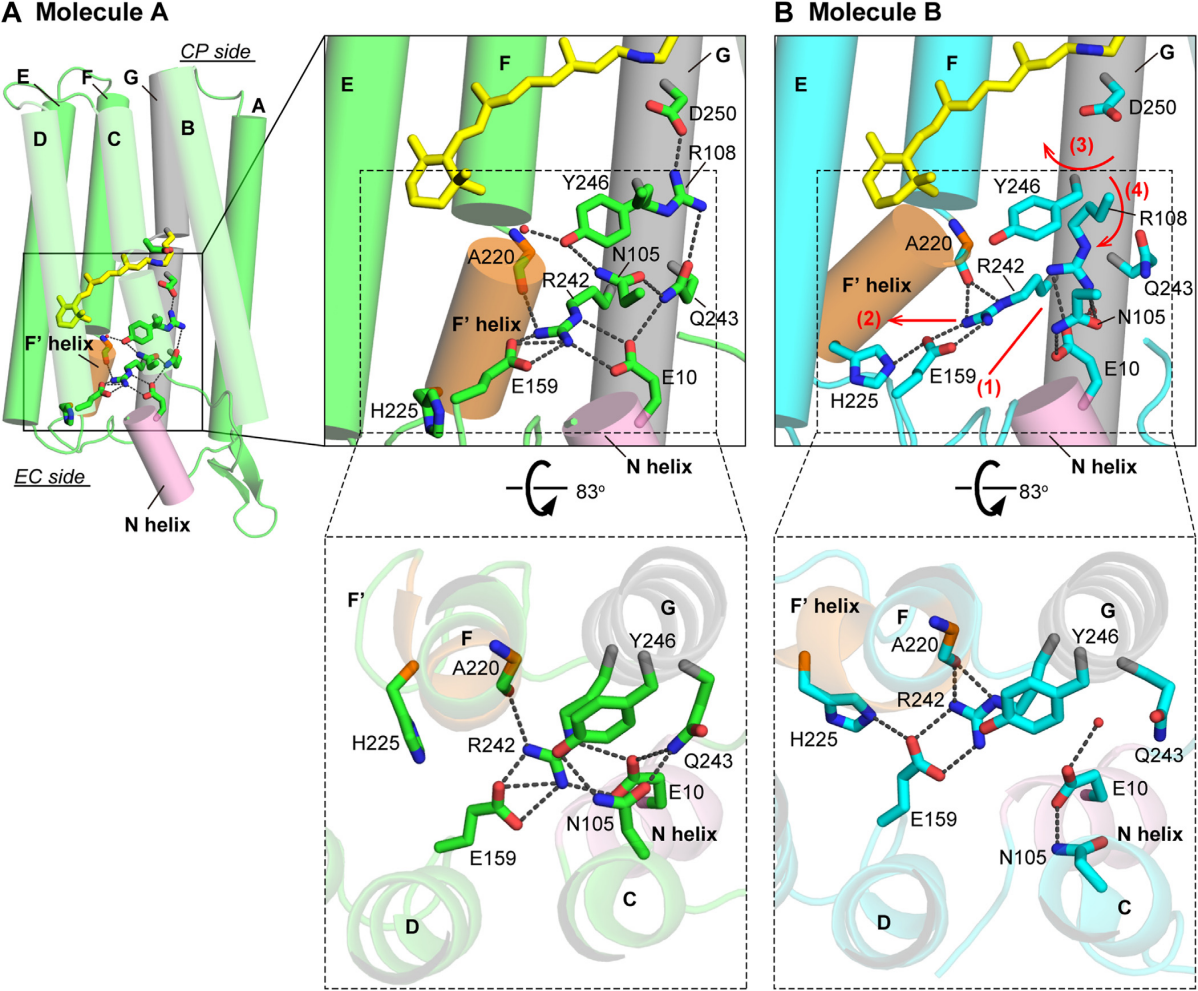
**Detailed comparison between the EC half structures of molecules A and B.***A*, the overall structure of molecule A is shown on the *left*, and its boxed area is enlarged in the *upper right panel*, whose area enclosed by the dotted line is rotated 83° and shown in the *lower panel*. *B*, the corresponding enlarged views of molecule B. Our hypothesis for the stepwise conformational changes is indicated in the *upper right panel* with numbered *red line* and *arrows*. For clarity, helices C and D are not shown in the *upper* enlarged panels. In the *lower* panels, retinal and Arg108 are not shown. The same panels are shown in Fig. S5 together with the superimposed images of molecules A and B. CP, cytoplasmic; EC, extracellular.

Figure 5 shows a detailed comparison between the EC half regions of molecules A and B. The same figure is shown in Fig. S5 together with their superimposed images. Our hypothesis is schematically shown in the upper panel of Figure 5*B* with the numbered red lines. In addition to the position of the Arg108 sidechain, the other notable feature of molecule B is the release of Glu10 residue from the three-residue cluster with Glu159 and Arg242 residues. Thus, we assumed that the photoinduced protein conformation change first disrupts the connection between Glu10 and the pair of Glu159 and Arg242 (the first step in Fig. 5*B*). This disruption might induce the outward movement of the pair and resultantly allow the hydrogen bonding interaction between Glu159 and His225 residues (the second step). Simultaneously, the hydrogen bond between the Arg242 sidechain (the other residue of the pair) and carbonyl oxygen of Ala220 becomes stronger than that in molecule A. Resultantly, the pair is stabilized in this outwardly open arrangement. Arg242 is located on the G helix, and thus, the G helix is slightly rotated by the movement of the Glu159-Arg242 pair (the third step) (Fig. S5*C*). On the G helix, Try246 is located one turn away from Arg242. In molecule A (Fig. 5*A*), the Tyr246 residue forms an extensive hydrogen bonding network involving Asn105, Gln243, and even Arg108. In molecule B (Fig. 5*B*), the G-helix rotation induces a displacement of Tyr246, which disrupts the hydrogen bonding network and resultantly induces the large displacements of these residues. For Asn105, its sidechain is inverted to the EC side. This displacement seems to open the space and then enable Arg108 to orient its sidechain downward (the fourth step). This tentative model for the sequential changes was examined by the following experiments.

### Can the "swing motion" be induced by a mutation?

**Figure 6:**
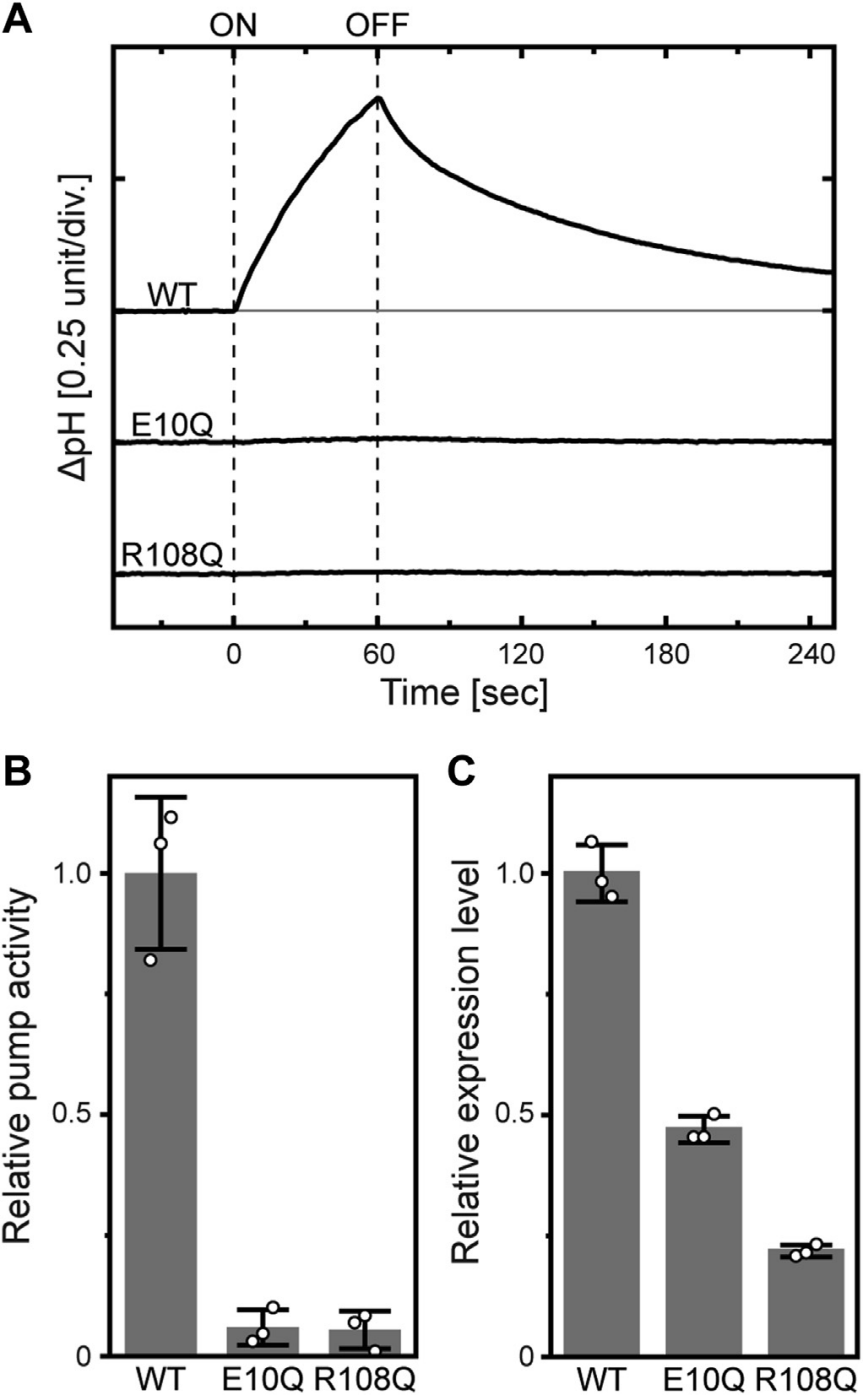
**Na**^**+**^**-pumping activities of IaNaR and the mutants.***A*, time courses of light-induced pH changes in the *Escherichia coli* suspensions. The illumination period is indicated with *vertical* broken lines. The *E. coli* cells were suspended in 200 mM NaCl solution containing 10 μM CCCP. *B*, the relative pumping activities. The initial slopes of the light-induced pH changes were divided by the respective expression levels. The resultant relative values are plotted here. *C*, the relative expression levels in the *E. coli* cell membranes. They were estimated from the maximum values of flash-induced absorbance changes at λ_max_ in the dark state. In (*B*) and (*C*), the scatter of *open circles* represents each measurement, and the bar represents the mean ± SD (n = 3). λ_max_, absorption maximum wavelength; CCCP, carbonyl cyanide m-chlorophenylhydrazone; IaNaR, *Inidibacter alkaliphilus* sodium pump rhodopsin.

We assumed that the "release" of the Glu10 residue from the cluster is the first step leading to the swing motion of Arg108. If this is the case, the swing motion of Arg108 can be driven even in the dark state by mutation of the Glu10 residue. Here, we replaced Glu10 with Gln to release it from the cluster. This mutation effect was examined by measuring the Na^+^-pumping activity. For Arg109 of KR2 (corresponding to Arg108 of IaNaR), its replacement by Gln is known to significantly distort the photoreaction and completely remove the Na^+^-pumping activity (2). If the swing motion occurs, the positive charge of the Arg residue becomes far from the original position. This situation should be similar to that of the R108Q mutant. Thus, the E10Q mutation might remove Na^+^-pumping activity. The experimental results are shown in Figure 6. Here, we measured the light-induced pH change of the *Escherichia coli* suspension. The outward Na^+^ transport creates an inside negative membrane potential, which induces passive H^+^ inflow and resultantly induces a pH increase. As shown in Figure 6*A*, WT IaNaR caused a distinct pH increase, whereas no pH change was observed for not only the R108Q but also the E10Q mutant. The initial slope of the light-induced pH change reflects the Na^+^-pumping activity. These slopes are plotted in Figure 6*B* after compensation of the expression levels, whose values are plotted in Figure 6*C*. As expected, the E10Q mutant exhibited only negligible activity, as did the R108Q mutant.

**Figure 7:**
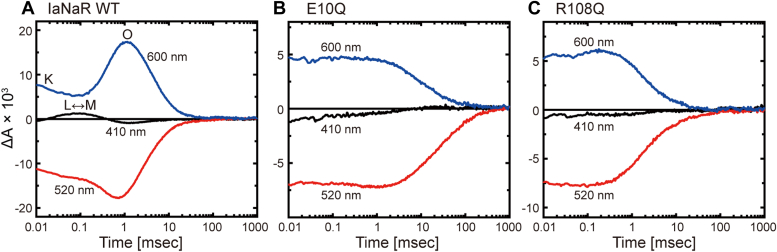
**Flash-induced absorbance changes of IaNaR at three typical wavelengths.** Panels (*A*–*C*) represent the data for (*A*) WT IaNaR, (*B*) E10Q, and (*C*) R108Q mutants, respectively. IaNaR, *Indibacter alkaliphilus* sodium pump rhodopsin.

Next, we examined the photocycles. The disabled E10Q and R108Q mutants should exhibit different photocycles than WT IaNaR. Here, we measured the flash-induced absorbance changes. As shown in Figure 7*A*, the photocycle of WT IaNaR involves multiple intermediates. The first one is K at 600 nm. Due to its fast formation, only the decay phase was observed even at 0.01 ms. Subsequently, the quasi-equilibrium between L and M appeared at 410 nm at approximately 0.1 ms. With their concomitant decay, the redshifted O appeared at 600 nm at approximately 1 ms. In contrast, for both the E10Q and R108Q mutants (Fig. 7, *B* and *C*), only a K-like intermediate appeared at 600 nm in the photocycle. These results support our model. In the E10Q mutant, the arginine-positive charge is far from its original position even in the dark state. As a result, E10Q exhibits a photocycle similar to that of the arginine-lacking R108Q mutant.

### Whether the "swing motion" can be induced at acidic pH?

**Figure 8:**
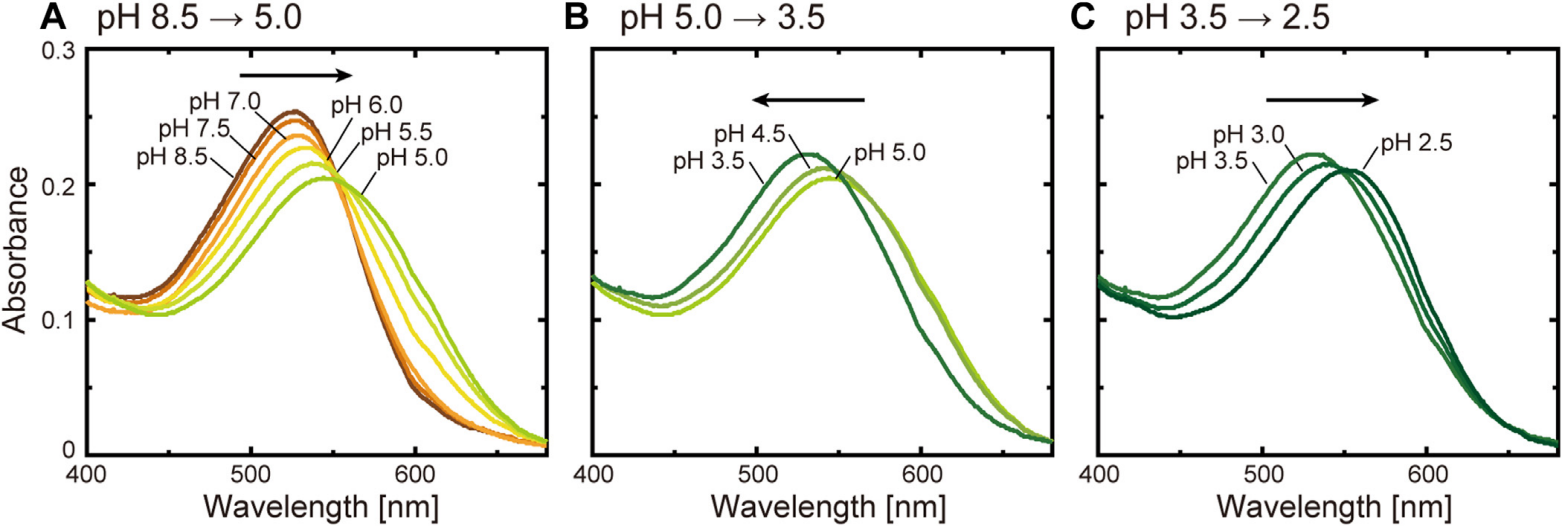
**pH-dependent spectral shifts of IaNaR.** The absorption spectra of lipid-reconstituted IaNaR were measured at various pH values. These spectra are plotted in (*A*–*C*), dividing the pH range into (*A*) pH 8.5 to 5.0, (*B*) pH 5.0 to 3.5, and (*C*) pH 3.5 to 2.5. IaNaR, *Indibacter alkaliphilus* sodium pump rhodopsin.

**Figure 9:**
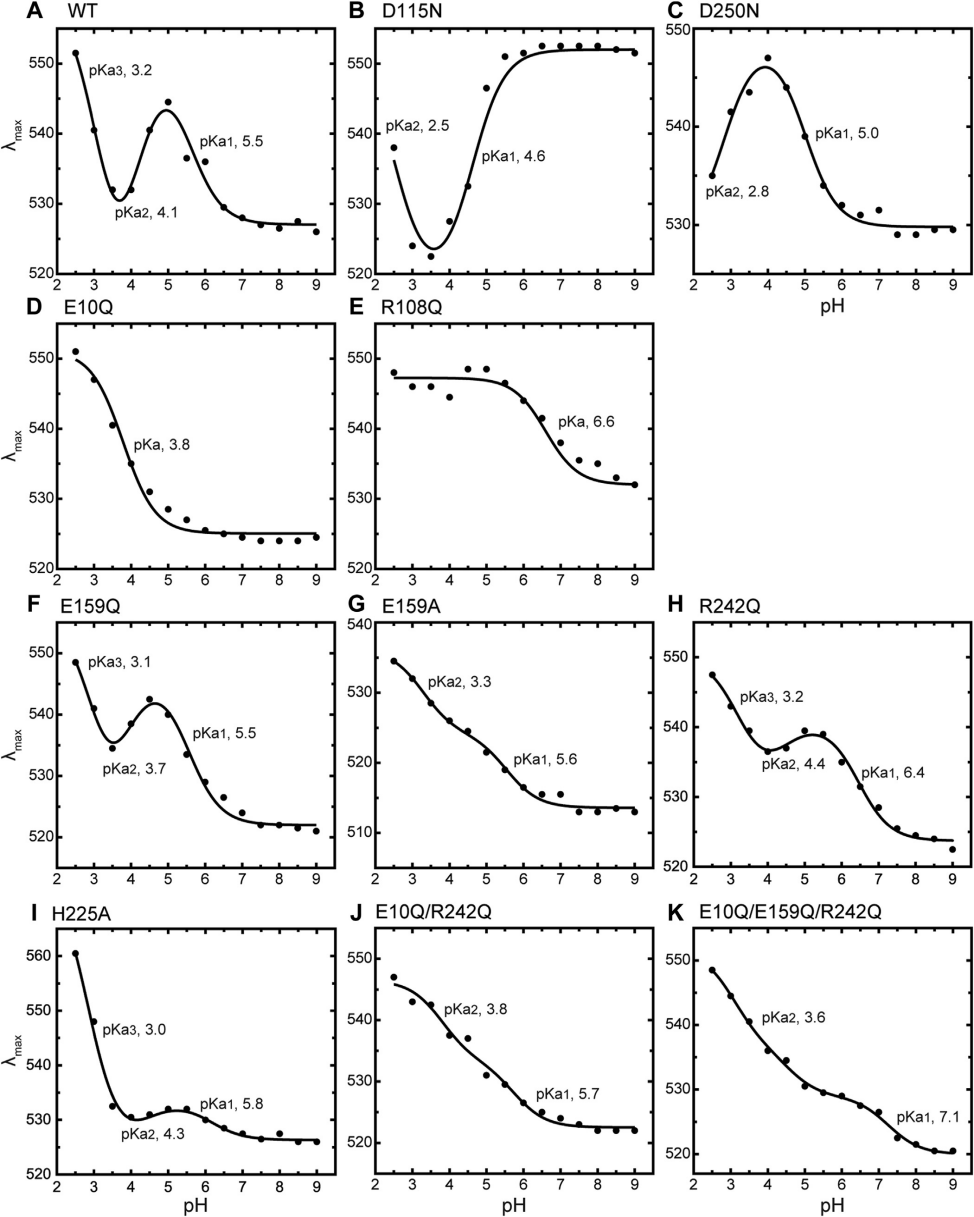
**pH-dependent λ**_**max**_**shifts of IaNaR and the mutants.** The λ_max_ values were depicted from the spectra and plotted with filled circles against pH. *Panels A*‒*K* represent the data for (*A*) WT IaNaR, for the single-replacement mutants (*B*) D115N, (*C*) D250N, (*D*) E10Q, (*E*) R108Q, (*F*) E159Q, (*G*) E159A, (*H*) R242Q, and (*I*) H225A and for the multiple-replacement mutants (*J*) E10Q/R242Q and (*K*) E10Q/E159Q/R242Q, respectively. The smooth lines indicate the best fitted results with Equation 1. The determined p*K*a values are shown in the respective panels. These panels are also shown in Fig. S6, along with the respective absorption spectra and those of KR2. λ_max_, absorption maximum wavelength; IaNaR, *Indibacter alkaliphilus* sodium pump rhodopsin; KR2, *Krokinobacter eikastus* rhodopsin 2.

In the WT protein, the Glu10 residue should have a negative charge and then interact with the pair of Glu159 and Arg242 residues. When the Glu10 residue is protonated at acidic pH, the interaction is probably disrupted, and the swing motion of the Arg108 residue seems to occur even in the WT protein. We tested this prospect by the pH dependence of the absorption spectrum. In the vicinity of the PSB, there are two negatively charged Asp115 and Asp250 residues and one positively charged Arg108 residue (Fig. 1*B*). When the Asp residues are protonated at acidic pH, the electronic ground state of retinal should become unstable, so the spectrum should be redshifted twice. In contrast, the swing motion of Arg108 removes its positive charge from the vicinity of PSB and should resultantly stabilize the electronic state of retinal. In this case, the opposite blueshift of the spectrum should occur. Figure 8 shows the pH-dependent spectral shifts of WT IaNaR. Here, we used lipid-reconstituted IaNaR, and thus, the spectra involve large scattering artifacts at shorter wavelengths. However, a complex spectral shift was clearly observed. By lowering the pH, the spectra initially shifted to longer wavelengths (Fig. 8*A*) but subsequently shifted to shorter wavelengths (Fig. 8*B*) and then finally shifted to longer wavelengths again (Fig. 8*C*). In Figure 9*A*, the absorption maximum wavelengths (*λ*_max_) are plotted against pH. The smooth line indicates the best-fitted curve using the following equation:

(1) $$\lambda_{\max}=\sum_{i}\frac{A_{i}}{1+10^{\text{pH}-{pKa}_{i}}}+\lambda_{\max,0}$$

where p*K*a_i_ represents the p*K*a values of Asp115, Asp250, and Glu10 residues, and *A*_i_ indicates the width of the spectral shift by the protonation of the respective residue. The term *λ*_max,0_ indicates λ_max_ at alkaline pH. As clearly shown in Figure 9*A*, by lowering the pH, the WT protein exhibited two redshifts with p*K*a values of 5.5 and 3.2 (p*K*a_1_ and p*K*a_3_), reflecting the protonation of two aspartic acid residues, Asp115 and Asp250. In addition, a blueshift also occurs with a p*K*a of 4.1 (p*K*a_2_), probably reflecting the protonation of the Glu10 residue and the resultant swing motion of the Arg108 sidechain. Consistent results were obtained for the replacement mutants of the Asp residues (Fig. 9, *B* and *C*). When Asp115 was replaced by Asn (Fig. 9*B*), the first redshift in the WT protein (p*K*a_1_, 5.5) disappeared. Thus, this redshift reflects the protonation of the Asp115 residue. When the other Asp250 was replaced by Asn (Fig. 9*C*), the second redshift in the WT protein (p*K*a_3_, 3.2) disappeared. Thus, this redshift probably reflects the protonation of the Asp250 residue. Next, to confirm the origin of the blueshift, we also examined the E10Q and R108Q mutants (Fig. 9, *D* and *E*). In both cases, only a single redshift was observed, and the blueshift disappeared. We expected that two redshifts would remain. Thus, at present, we cannot clearly explain why the redshift occurred only once. However, the disappearance of the blueshift in both mutants is consistent with our hypothesis. For the E10Q mutant, the three-residue cluster is already disrupted, so the arginine sidechain should be oriented downward at any pH. Thus, this mutant did not exhibit a blueshift. For the R108Q mutant, the swing motion probably occurs similarly to WT IaNaR. However, this residue has no positive charge. Thus, the swing motion cannot lead to the blueshift. The pH-dependent spectral shifts of WT IaNaR and all mutants are summarized in Fig. S6.

### Subsequent processes after the disruption of the "cluster"

We assumed that after the release of the Glu10 residue from the cluster, Arg242 moves outward together with Glu159 (the second step in Fig. 5*B*). This movement probably induces the small rotation of the G-helix (the third step in Fig. 5*B*), which causes large displacements of several residues, such as Asn105 and Gln243, and finally induces the swing motion of the Arg108 sidechain (the fourth step in Fig. 5*B*). The outwardly open arrangement of Arg242 seems to be stabilized by its sidechain interactions with the carbonyl oxygen of Ala220 and the sidechain of Glu159. The latter also interacts with the His225 sidechain. When these interactions are disrupted by mutations, the swing motion of Arg108 should not occur even though the Glu10 residue is released from the cluster. To test this hypothesis, we made single mutants of E159Q, E159A, R242Q, and H225A and multiple mutants of E10Q/R242Q and E10Q/E159Q/R242Q. The pH dependences of the spectral shifts are shown in Figure 9, *F*–*K*. Of the single mutants, E159Q, R242Q and H225A (Fig. 9, *F*, *H* and *I*) still exhibited blueshifts. Thus, the swing motion still occurs by protonation of the Glu10 residue. However, the shift widths are small, especially for R242Q and H225A (Fig. 9, *H* and *I*). Thus, the open arrangement of the 242nd residue might become unstable. Of note, the blueshift disappeared for the E159A mutant (Fig. 9*G*), where only two redshifts were observed. This result supports our prospect. Even in the E159A mutant, the Glu10 residue should become protonated at acidic pH, and its release from the cluster might occur. However, this release cannot lead to the movement of the Arg242 residue and resultant swing motion of the Arg108 sidechain.

Consistent results were also obtained in the multiple mutants E10Q/R242Q and E10Q/E159Q/R242Q (Fig. 9, *J* and *K*). In both mutants, the Glu10 residue is also replaced by Gln. As shown above, the E10Q single mutation enforced the downward orientation of the Arg108 sidechain at any pH and resulted in a significantly distorted pH profile (Fig. 9*D*), where only a single redshift appeared. In contrast, significantly different pH profiles were observed for the multiple mutants. Both mutants exhibited two redshifts (Fig. 9, *J* and *K*), suggesting that the Arg108 sidechain is oriented upward at any pH. By breaking the interaction around the 242nd residue, the E10Q mutation probably lost the ability to cause displacement of the 242nd residue and subsequent conformational changes. Thus, the outward movement of the Arg242 residue appears to be a key process leading to the swing motion of the Arg108 residue.

### Do sequential changes actually occur during the photocycle?

**Figure 10:**
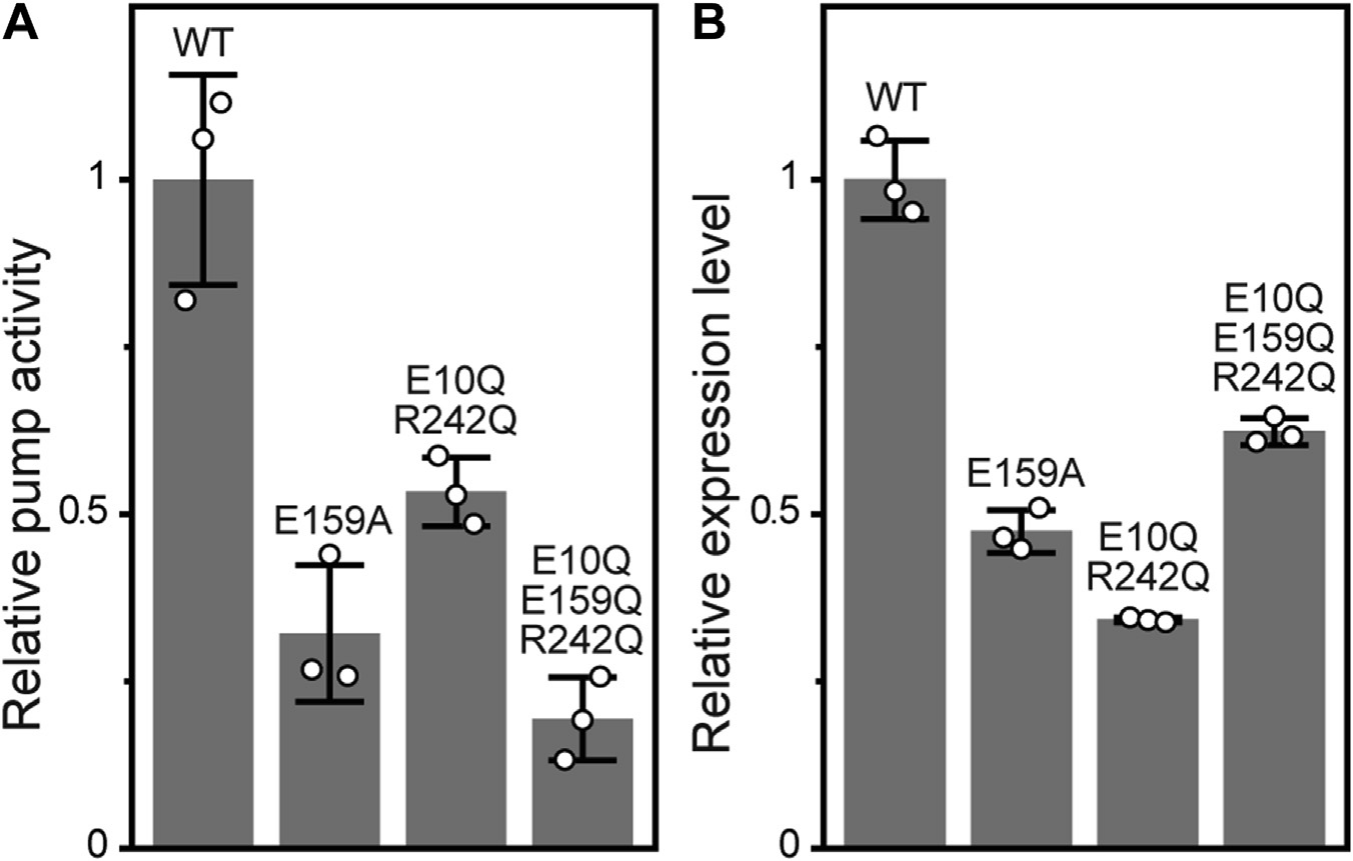
**Comparison of Na**^**+**^**-pumping activities of IaNaR and the mutants lacking acid-induced blueshift.** The experimental procedures are the same as those of Figure 6. The relative pumping activities (*A*) and the relative expression levels in the *Escherichia coli* cell membranes (*B*) are shown here. The scatter of *open circles* represents each measurement, and the bar represents the mean ± SD (n = 3). The time courses of light-induced pH changes are shown in Fig. S7, along with the corresponding data for all samples tested in this study. IaNaR, *Indibacter alkaliphilus* sodium pump rhodopsin.

The important question is whether the predicted sequential changes actually occur during the photocycle. As mentioned above, the swing motion of Arg108 probably facilitates Na^+^ translocation. This swing motion should not occur for the mutants of E159A, E10Q/R242Q, E10Q/E159Q/R242Q because these mutants did not show the spectral blueshift by lowering pH. Thus, they cannot cause outward displacement of the 242nd residue and are unlikely to drive the subsequent conformational changes during the photocycle. To test the functional importance of the sequential changes, we measured the Na^+^-pumping activities of those mutants. Figure 10*A* shows their relative pumping activities after accounting for the difference in respective protein expression levels, whose values are plotted in Figure 10*B*. As shown here, all mutants exhibited distinct activity even though the "automated system" for sequential conformation changes is probably broken. Thus, this system might not be essential for Na^+^-pumping function. However, all mutants exhibited significantly reduced activities, suggesting that the strong activity of the WT protein is conferred by the sequential conformation changes leading to the swing motion of the Arg108 residue. These data are shown again in Fig. S7, along with the time courses of light-induced pH changes.

As mentioned above, the E10Q single mutant exhibited only negligible activity (Fig. 6), which is a clear contrast with the distinct activities of E10Q/R242Q and E10Q/E159Q/R242Q (Fig. 10). Thus, the additional mutations of R242Q and E159Q/R242Q restore pumping activity, probably restoring the upward orientation of Arg108 in the resting dark state. The restoration of the activity should occur together with the restoration of the photocycle. Fig. S8 summarizes the flash-induced absorbance changes of the WT IaNaR and the mutants whose Na^+^-pumping activities were measured in this study. As mentioned above, the E10Q mutant exhibited a significantly distorted photocycle (Figs. 7*B* and S8*B*), where only a K-like intermediate appeared. As shown in Fig. S8*E*, the additional mutation of R242Q surely restores the photocycle, where K, L/M, and O intermediates clearly appear, although the accumulation of O is somewhat smaller, and the O decay is slower than that of WT IaNaR (Figs. 7*A* and S8*A*). In contrast, the triple mutants (E10Q/E159Q/R242Q) still exhibited distorted photocycles (Fig. S8*F*), where the O intermediate seemed to appear slightly at approximately 5 ms. Its accumulation is faint but might be important for pumping activity. In addition to these mutants, E159A showed reduced Na^+^-pumping activities (Fig. 10), which might reflect only slight accumulations of L/M and O intermediates in its photocycle (Fig. S8*D*).

The disruption of the "automated system" is likely to slow down the Na^+^ release during the O decay. Indeed, the E10Q/R242Q mutant exhibited slower O decay as mentioned above (Fig. S8*E*). This trend was also observed in other mutants of E159A and E10Q/E159Q/R242Q (Fig. S8, *D* and *F*), although their O accumulations are small. To compare the O decay rate, we analyzed the data of WT IaNaR and the E10Q/R242Q mutant. The analysis results are summarized in Fig. S9. Here, we simultaneously fitted the data at three wavelengths (410, 520, and 600 nm) according to the sequential irreversible model: P0 → P1 → P2 → ⋅⋅⋅ → Pn → P0, where P0 represents the original dark state, and P*i* (*i* = 1, 2, ⋅⋅⋅, n) represents the kinetically distinguishable states, respectively (21) (for details, see Supplementary Experimental Procedures). Fig. S9, *A* and *B* indicate the fitting results for n = 6. Thus, both panels also show the calculated concentration changes of the six P states (black and green lines). In both fitting results, O accumulations at approximately 0.03 ms to 30 ms are described by the P4 and P5 states, reflecting that O is divided into two substates, O1 and O2, as mentioned above. Thus, the O2 decay (*i.e.*, P5 decay) corresponds to the Na^+^-releasing step. Interestingly, the decay time constants preceding O2 decay (*τ*_1_-*τ*_4_) are almost the same between WT IaNaR and the mutant. However, with respect to O2 decay, the time constant (*τ*_5_) of the mutant (13 ms) is 2.4 times slower than that of the WT IaNaR (5.5 ms), indicating that the disruption of the "automated system" does indeed delay Na^+^ release. In addition to the O2 decay, the last step (*τ*_6_) is also slower in the mutant (192 ms) as compared to that of the WT IaNaR (110 ms). During this step, only small absorption changes occur. Thus, there is no essential difference in the chromophore structure and its surrounding environment between P6 and the dark state. Instead, the structure of the other parts is probably different. The "automated system" also contributes to this final restoration of the protein structure, which should mainly occur at the Na^+^ release side.

## Discussion

In this study, we solved the tertiary structure of IaNaR, which inspired us to consider the sequential conformation changes leading to the gate-open state for Na^+^ release. Herein, we assumed that the Arg108 sidechain acts as a gate and that its downward orientation corresponds to the gate-open state. The following experiments almost supported our model, but most data were obtained for the unphotolyzed states. Thus, further study is necessary to clarify whether sequential changes occur during the photocycle. Nevertheless, our experiments indicated that at least in the unphotolyzed IaNaR, the swing motion of the Arg108 residue is induced through multiple conformational changes, which can be triggered by only the release of the Glu10 residue from the cluster at the EC surface. This cluster is conserved in many NaRs and is known to elevate the structural stability from the analysis using KR2 (16). In addition to its role in stability, this cluster might be important for maintaining the upward orientation of the Arg residue (Arg108 of IaNaR and Arg109 of KR2) until Na^+^ release in the latter half of the photocycle. If the Arg sidechain orients downward even in the dark state, NaR cannot undergo the Na^+^-pumping photocycle.

### The importance of the swing motion of the Arg residue

As mentioned above, the Arg sidechain (Arg108 of IaNaR; Arg109 of KR2) seems to block the Na^+^ translocation pathway in the dark state. Thus, the swing motion seems to be necessary to release Na^+^. This view was previously shown by an MD simulation for KR2 (22), where the Arg sidechain orients downward when the Na^+^ moves to the EC side from the vicinity of Asp251. Similar swing motion was also observed in the time-resolved X-ray crystallography for KR2, as mentioned above (12). In the 20 ms structure (Fig. S1*D*), Na^+^ disappears around Asp251. Simultaneously, the Arg109 sidechain is oriented downward, suggesting that the swing motion occurs before or simultaneously with the Na^+^ movement to the EC side. Moreover, a previous study on the R109Q mutant of KR2 indirectly supported swing motion in the WT protein (23). This mutant exhibited significantly reduced Na^+^-pumping activity, as mentioned above, and simultaneously showed passive potassium transport. Thus, the Arg sidechain surely acts as the gate, and its removal imparted channel-like properties. These data support that, in the WT protein, the swing motion of the Arg sidechain might allow Na^+^ translocation during the photocycle.

### Do sequential conformational changes also occur in KR2?

For IaNaR, a complex pH dependence of the absorption spectrum was observed, as shown in Figures 8 and 9*A*. This complex shift fit our hypothesis well. In particular, the spectral blueshift is notable because this shift probably reflects the swing motion of the Arg108 sidechain triggered by protonation of the Glu10 residue. However, for KR2 and another NaR from the eubacterium *Gillisia limnaea* (GLR), only a monotonous redshift was reported (2, 24). Their lipids for reconstitution were different from our samples. Thus, we examined the spectral shift for KR2 with the same conditions for IaNaR. However, a monotonous redshift was also observed, as shown in Fig. S6*L*. In addition to the spectral shift, a characteristic difference between IaNaR and KR2 also exists in the mutation effect of E10Q IaNaR and the corresponding E11Q KR2. For E10Q IaNaR, we observed only negligible Na^+^-pumping activity (Fig. 6). In contrast, E11Q KR2 was reported to exhibit almost the same Na^+^-pumping activity as WT KR2 (16). Thus, KR2 and GLR might not have the "automated system", eventually leading to the swing motion of the Arg sidechain. However, even for KR2 and GLR, this swing motion seems to be necessary for Na^+^ release. How do they achieve the swing motion during the photocycle? The same question arises for the IaNaR mutants E159A, E10Q/R242Q, and E10Q/E159Q/R242Q. As shown in Figures 9 and 10, these mutants lacked the automated system but still exhibited Na^+^-pumping activity. A possible driving force for their swing motion might be the approaching Na^+^ itself. As mentioned above, Na^+^ binds to the vicinity of Asp250 (corresponding to Asp251 of KR2 and GLR). The resultant repulsive force might push the Arg sidechain to induce the swing motion. For the IaNaR mutants, we observed reduced Na^+^-pumping activities and slower decays of O intermediates during their photocycles. Thus, the simple repulsive force by Na^+^ might induce only a small swing motion of the Arg sidechain and might resultantly slow down the Na^+^-releasing process during the O decay. In contrast, the "automated system" drives the large swing motion, which might facilitate the Na^+^ release and resultantly confer strong Na^+^-pumping activity. According to this prospect, WT IaNaR should exhibit faster O decay and stronger Na^+^-pumping activity than KR2 and GLR. Thus, an accurate comparison of their photocycles and transport activities might be an interesting issue for future investigation.

### What is the mechanism for light-induced release of Glu10 residue from the cluster?

Our hypothesis assumes that the release of the Glu10 residue occurs first, and all subsequent steps are induced stepwise. Although this model is consistent with our data, it might be too simple to describe the events in the protein, especially the release of the Glu10 residue. The cluster is located on the EC surface and is far from the retinal, that is, the reaction center of this protein. The conformational change at such a protein surface seems to occur through multiple steps that propagate from inside to outside of the protein. Thus, the release of the Glu10 residue might be caused by the collaboration of several reactions. One of them might be the approaching Na^+^, which was already assumed for the mechanism of swing motion when the automated system is absent. Similarly, the approaching Na^+^ in the WT protein might induce a slight swing motion of the Arg108 residue, which can push the positively charged Arg242 to some extent. This displacement might eventually cause the release of the Glu10 residue, which might allow large outward movement of the Glu159-Arg242 pair and then induce subsequent reactions. In addition to this scenario, Na^+^ binding to the cluster itself might assist in the release of the Glu10 residue. This Na^+^ binding was observed in the structure of the last intermediate determined by time-resolved X-ray crystallography (Fig. S1*D*). This Na^+^ might come from the EC medium and then facilitate the release of Glu10 residue.

In molecule B, the F' helix largely bends outward. Due to this bending, the Cα atom of His225 outwardly moves 3.8 Å from its corresponding position in molecule A (Fig. 5). The His225 sidechain forms a hydrogen bond with Glu159 and appears to stabilize the open arrangement of the Glu159-Arg242 pair. However, the bending of the F' helix is caused by an unnatural interaction with the neighboring protomer (Fig. S4), indicating that large bending should not occur in the lipid membrane. Why can the Glu159-Arg242 pair take an open arrangement and then lead to subsequent conformational changes? In addition to the His225–Glu159 interaction, the position of the Glu159-Arg242 pair is also stabilized by the interaction between the Arg242 sidechain and the carbonyl oxygen of Ala220, which is located just above the F' helix. Different from His225, this carboxyl oxygen is only 1.6 Å away from the corresponding position in molecule A. Thus, large bending of the F' helix might not be necessary to stabilize the open arrangement of the Glu159-Arg242 pair. Although we do not have a clear model, a small displacement of Ala220 might occur without the help of large bending of the F′ helix and then drive the subsequent conformational changes.

As mentioned above, the Asp115 residues in the dimer of our crystal are probably protonated. Protonation of this residue is also considered to occur during the formation of M intermediate, which appears before the O intermediate during the photocycle. Thus, it is interesting to consider the relationship between the protonation of Asp115 and the swing motion of Arg108. In the case of archaeal H^+^ pump bacteriorhodopsin, protonation of an Asp residue (Asp85) corresponding to Asn111 of IaNaR is known to induce the swing motion of Arg82, corresponding to Arg108 of IaNaR (25). This Asp-Arg coupling of bacteriorhodopsin is observed not only in the photolyzed state but also in the unphotolyzed state. Most of the data in the present study were obtained in the unphotolyzed state. These data seem to rule out the strong Asp-Arg coupling, at least in the dark state. As shown in Figure 9*A*, the spectral blueshift reflecting the swing motion occurred around pH 4 (p*K*a, 4.1). This pH region is clearly different from that of the protonation of Asp115 (p*K*a, 5.5). If strong coupling is present, two pH regions should overlap. Moreover, the strong coupling is also denied by the D115N data (Fig. 9*B*), where the blueshift (*i.e.*, the swing motion of Arg108 residue) was still observed (p*K*a, 4.6) even though the negative charge at 115th position was already removed by the mutation. These results are consistent with a report on the D116N KR2 mutant (11). In the crystal structure of this mutant, the Arg109 sidechain was revealed to maintain the upward orientation. Thus, the conformational change for Na^+^ release cannot be caused by the protonation of the Asp residue at least in the dark state. This conformational change needs other mechanisms as described above.

## Conclusion

Most proteins require sequential conformation changes to exhibit their respective functions. Understanding how proteins drive such multistep reactions is a fascinating research topic. In this study, we solved the IaNaR dimer structures, which led us to examine the mechanism of conformational changes. The results suggested that the sequential conformation changes are triggered by the disruption of three residue cluster at the EC surface and finally lead to the gate-open state. KR2, the representative NaR, might not have the same system but should have an alternative system to drive the swing motion of the Arg109 sidechain. Even in this alternative system, cluster disruption might be important. As mentioned above, the cluster-lacking mutants of KR2 are known to have significantly weak structural stability (16), probably reflecting the flexible conformation at the EC half side. Higher flexibility should be important to induce the swing motion of the Arg sidechain. Thus, cluster disruption might be the common mechanism for photolyzed NaRs to eventually reach the gate-open state. Most experiments in this study were performed in the dark. Thus, it is an open question whether similar structural changes surely occur during photoreactions. The elucidation of this issue is an interesting subject for future investigation.

## Experimental procedures

### Gene expression and protein purification

*E. coli* DH5α was used for DNA manipulation. The expression plasmid of IaNaR was described previously, where the IaNaR gene was inserted into the pKA001 vector under the lacUV5 promoter (13, 26). The expressed IaNaR has a six-histidine tag in the C terminus. Mutations were introduced using the QuikChange Site-Directed Mutagenesis Kit (Agilent Technologies). The DNA sequences were confirmed by a standard procedure.

*E. coli* BL21 was used for the expression and purification of IaNaR. The procedure was essentially the same as previously described (27). Briefly, the cells were grown in 2× YT medium at 37 °C, and expression was induced in the presence of 10 μM all-*trans* retinal by the addition of 1 mM IPTG. After 3 h of induction, the cells were harvested and broken with a sonicator. The collected cell membranes were solubilized with 1.5% n-dodecyl-β-D-maltopyranoside (DDM). The solubilized protein was purified using nickel-nitriloacetic acid agarose. For crystallization, the protein was further purified by gel-filtration chromatography (Superdex 200) in the presence of 50 mM Tris–HCl (pH 7.0), 50 mM NaCl, and 0.01% DDM. The purified protein was concentrated to 20 to 40 mg/ml using a centrifugal filter device (Millipore 30 kDa Mw cutoff) and frozen until crystallization.

### Crystallization, data collection, and structure determination

The purified IaNaR protein was mixed with monoolein (1-oleoyl-rac-glycerol) (Nu-Chek) in a 2:3 protein to lipid ratio (w/w). Aliquots (50–100 nl) of the protein-LCP mixture were spotted on a 96-well sandwich plate and overlaid with 800 to 1000 nl of precipitant solution. The initial crystals were obtained within 2 weeks at 20 °C in reservoir solution containing 0.1 M ammonium nitrate, 0.1 M sodium citrate (pH 5.0), and 30% (w/v) PEG350 MME and optimized with 0.1 M ammonium nitrate, 0.1 M sodium citrate (pH 5.1), and 25% PEG350 MME. The crystals were harvested directly from the LCP matrix and flash-cooled under a stream of liquid nitrogen at 100 K.

The X-ray diffraction dataset was collected on beamline BL-41XU at SPring-8 under a stream of liquid nitrogen at 100 K. The dataset was indexed, integrated, scaled, and merged using the *XDS* program suite (28). The asymmetric unit of IaNaR contained two molecules corresponding to a Matthews coefficient (29) of 2.29 Å^3^ Da^−1^ and an estimated solvent content of 46.3%. The statistics of the data collection and processing are summarized in Table S1.

The structure of IaNaR was determined by the molecular replacement method with the program *AutoMR* in the *Phenix* program package (30, 31) using the structure of KR2 (PDB: 3X3C) as a search model. Several rounds of refinement were performed using the program *Phenix.refine* in the *Phenix* program suite, alternating with manual fitting and rebuilding based on 2*F*_*o*_ − *F*_*c*_ and *F*_*o*_ − *F*_*c*_ electron density using *COOT* (31, 32). The final refinement statistics and geometry defined by MolProbity (33) are shown in Table S1. All structure figures were generated by PyMol (http://www.pymol.org/).

### Measurement of absorption spectra at various pH values

The purified IaNaR was reconstituted into the lipid membrane as previously described (34). Briefly, phosphatidylcholine from egg yolk (Avanti) was mixed at a protein:lipid molar ratio of 1:50. The detergent DDM was removed at 4 °C by gentle shaking in the presence of SM2 Adsorbent Bio-Beads (Bio-Rad). After filtration, the reconstituted proteins were harvested by centrifugation. These protein samples were easily precipitated and were not suitable for spectral measurements. Thus, they were resuspended in salt-free buffer and then encapsulated into a 15% acrylamide gel as previously described (35). For each sample, 14 to 17 gels were prepared. Their buffer solutions were replaced by immersing them into 6-mix buffer (citric acid, 5.48 mM; ADA, 1.84 mM; MOPS, 4.16 mM; TAPS, 5.00 mM; CHES, 3.24 mM; CAPS, 5.56 mM) containing 0.4 M NaCl with an appropriate pH. The absorption spectra were measured by a UV1800 spectrometer (Shimadzu) at room temperature.

### Flash photolysis spectroscopy

Flash-induced absorbance changes of IaNaR were measured by a single-wavelength kinetic system, whose details were described previously (36, 37). The actinic light was a second harmonic of the Q-switched Nd-YAG laser (532 nm, 5 ns, 1 mJ). At selected wavelengths, the subsequent absorbance changes were recorded and averaged by using 30 laser pulses to improve the S/N ratio. For the samples, *E. coli* cell membrane fragments expressing IaNaR were used. After disruption of the cells, the membrane fragments were collected as described above. These fragments were resuspended in aqueous solution and encapsulated in 15% acrylamide gel to prevent precipitation. The buffer solution was replaced with 6-mix buffer at pH 8.5 containing 0.4 M NaCl as described above. All measurements were performed at 25 °C.

### Sodium pump activity measurements

The outward Na^+^-pumping activities were measured by the conventional pH electrode method, which detects the pH increase of the *E. coli* suspension due to IaNaR activity. The procedures were essentially the same as those previously reported (38). Briefly, the cells expressing IaNaR were harvested and washed twice with 200 mM NaCl. The cell suspensions were gently shaken overnight at 4 °C in the presence of 10 μM carbonyl cyanide m-chlorophenylhydrazone. The next day, the cells were washed twice in the same solution and finally suspended in a 10 ml volume at an absorbance of 2.0 at 660 nm. After the addition of 10 μM carbonyl cyanide m-chlorophenylhydrazone, the suspensions were illuminated by green LED light at 530 ± 17.5 nm (LXHL-LM5C; Philips Lumileds Lighting Co). The light intensity was adjusted to approximately 24 mW/cm^2^ at 530 nm using an optical power meter (Orion-PD; Ophir Optronics Ltd).

The initial slopes of the pH changes reflect the pumping activities. However, these slopes also depend on the expression amounts of IaNaR. Thus, the expression levels were evaluated by the signal amplitudes of the flash-induced absorbance changes. After the measurements of light-induced pH changes, the *E. coli* cells were harvested and disrupted by sonication after resuspending in 25 mM MES, pH 6.5, containing 0.3 M NaCl. These cell lysates were used for the samples, and the flash-induced absorbance changes were measured at the respective λ_max_ in the dark state. To improve the S/N ratio, the absorbance changes were averaged 100 times. The negative deflected signals reached maximum values within 3 ms after flash excitation. These maximum values were utilized for the relative expression amounts of IaNaR. The relative pumping activities were calculated by dividing the initial slopes of pH changes by the maximum values of the flash-induced absorbance changes.

## Data availability

All of the data supporting the findings of this study are available within the paper and the supporting information. Atomic coordinates and structure factors for the reported structure of IaNaR have been deposited in the Protein Data Bank under accession code 8JH0. The PDB DOI is 10.2210/pdb8jh0/pdb.

## Supporting information

This article contains supporting information (8, 9, 17, 21).

## Conflict of interest

The authors declare that they have no conflicts of interest with the contents of this article.
