# Supplementary material for: Multistep conformational changes leading to the gate opening of light-driven sodium pump rhodopsin
Source: J Biol Chem. 2023 Oct 27;299(12):105393. doi: 10.1016/j.jbc.2023.105393 (PMC10679507; doi:10.1016/j.jbc.2023.105393)
Supplement: Supporting Information [file mmc1.pdf]

Supporting Information

Multistep conformational changes leading to the gate opening of light-driven sodium pump rhodopsin

Yukino Sato[#,1], Tsubasa Hashimoto[#,1], Koji Kato[2], Akiko Okamura[1], Kaito Hasegawa[1], Tsukasa Shinone[1], Yoshikazu Tanaka[3], Yoshiki Tanaka[4], Tomoya Tsukazaki[4], Takashi Tsukamoto[1,5], Makoto Demura[1,5], Min Yao[1,5], Takashi Kikukawa[*,1,5]

[1] Graduate School of Life Science, Hokkaido University, Sapporo 060-0810, Japan

[2] Research Institute for Interdisciplinary Science, Okayama University, Okayama 700-8530, Japan

[3] Graduate School of Life Sciences, Tohoku University, Sendai 980-8577, Japan

[4] Graduate School of Biological Sciences, Nara Institute of Science and Technology, Nara 630-0192, Japan

[5] Faculty of Advanced Life Science, Hokkaido University, Sapporo 060-0810, Japan

[#] These authors contributed equally to this work.

[*] Corresponding author

Dr. Takashi Kikukawa, E-mail: kikukawa@sci.hokudai.ac.jp.

Table of Contents

Supplementary Experimental Procedures S3

Fitting analysis of the flash-induced absorbance changes S3

Supplemental Table S4

Table S1. Summary of crystallization conditions, data collection and refinement statistics.......... S4

Supplemental Figures S5

Figure S1. Tertiary structures of KR2.......... S5

Figure S2. KR2 structure in the dark state.......... S6

Figure S3. Comparison of CP side structures among two IaNaR protomers and KR2.......... S7

Figure S4. Inter protomer interaction of IaNaR.......... S8

Figure S5. Detailed comparison between the EC half structures of molecule A and B.......... S9

Figure S6. pH-induced absorption spectral shifts of all samples tested in this study.......... S10

Figure S7. Na^+ pumping activities of all samples tested in this study.......... S12

Figure S8. Flash-induced absorbance changes of all samples tested in this study.......... S13

Figure S9. Analysis results of the flash-induced absorbance changes S15

Supplementary Experimental Procedures

Fitting analysis of the flash-induced absorbance changes

The flash-induced absorbance changes at three typical wavelengths (410, 520, and 600 nm) were analyzed according to the sequential irreversible model (21), which describes the photocycle by one-way transitions of the kinetically distinguishable states, Pi (i=1, 2, ..., n). The details of the analysis procedure are described previously (9). According to this model, the flash-induced absorbance change at each wavelength, $\Delta A(t, \lambda)$, is described by the following multiexponential function:

$$\Delta A(t,\lambda) = \sum_{i=1}^{n} A_i(\lambda) \exp\left(-\frac{t}{\tau_i}\right)$$

where τ_i denotes the decay time constant of Pi state and is not dependent on the measuring wavelength. Thus, τ_i is the global parameter in the fitting analysis. The number of exponents correspond to the number of Pi states (n). To determine this number, the fitting analysis was performed by varying the number of exponents. Then, the results were evaluated by the standard deviations of the weighted residuals. Prior to the fitting analyses, the noise level at each wavelength was estimated by calculating the standard deviation of the data over the time range of -40 to 0 msec. The reciprocals of the standard deviations were used as weights to calculate the fitting residuals, so that the standard deviations of the fitting residuals are close to 1 at any wavelengths if the best fitting results are obtained. By using the determined time constants, the concentration changes of the Pi states are calculated by the following equations:

$$P_i(t) = \sum_{j=1}^{i} a_{i,j} \cdot \exp\left(-\frac{t}{\tau_j}\right)$$

where

$$a_{i,j} = 1 \Big/ \prod_{m=1}^{i-1} \tau_m \prod_{\substack{m=1 \\ m \neq j}}^{i} \left(1/\tau_m - 1/\tau_j\right)$$

The P$i(t)$ takes values from 0 to 1.

Supplemental Table

Table S1. Summary of crystallization conditions, data collection and refinement statistics.

	IaNaR
PDB ID	8JH0
Data collection	
Beamline	SPring-8 BL41XU
Space group	$P2_12_12_1$
Unit cell parameters *a*, *b*, *c*, (Å)	67.4, 71.8, 123.9
Wavelength (Å)	1.0
Resolution range (Å)	50.0-2.8 (2.96-2.8)
R_{meas} (%)	13.4 (80.5)
$\langle I/\sigma(I)\rangle$	13.6 (2.1)
Completeness (%)	98.1 (89.5)
Redundancy	6.3 (5.0)
Refinement	
No. reflection	15200
R_{work}/R_{free} (%)	24.3/28.7
No. of atoms	
Macromolecules	4262
Water	20
B-factors ($Å^2$)	
Macromolecules	58.4
Water	44.1
Estimated coordinate error	0.38
Rmsd from ideal	
Bond lengths (Å)	0.004
Bond angles (°)	0.686

Values in parentheses are for the highest resolution shell.

$^{*}R_{meas} = \Sigma_{hkl} \{N(hkl)/[N(hkl) - 1]\}^{1/2} \Sigma_i | I_i(hkl) - \langle I(hkl)\rangle | / \Sigma_{hkl} \Sigma_i I_i(hkl)$, where $\langle I(hkl)\rangle$ and $N(hkl)$ are the mean intensity of a set of equivalent reflections and the multiplicity, respectively.

$^{**}R_{work} = \Sigma_{hkl} ||F_{obs}| - |F_{calc}||/\Sigma_{hkl} |F_{obs}|$, R_{free} was calculated for 5% randomly selected test sets that were not used in the refinement.

Supplemental Figures

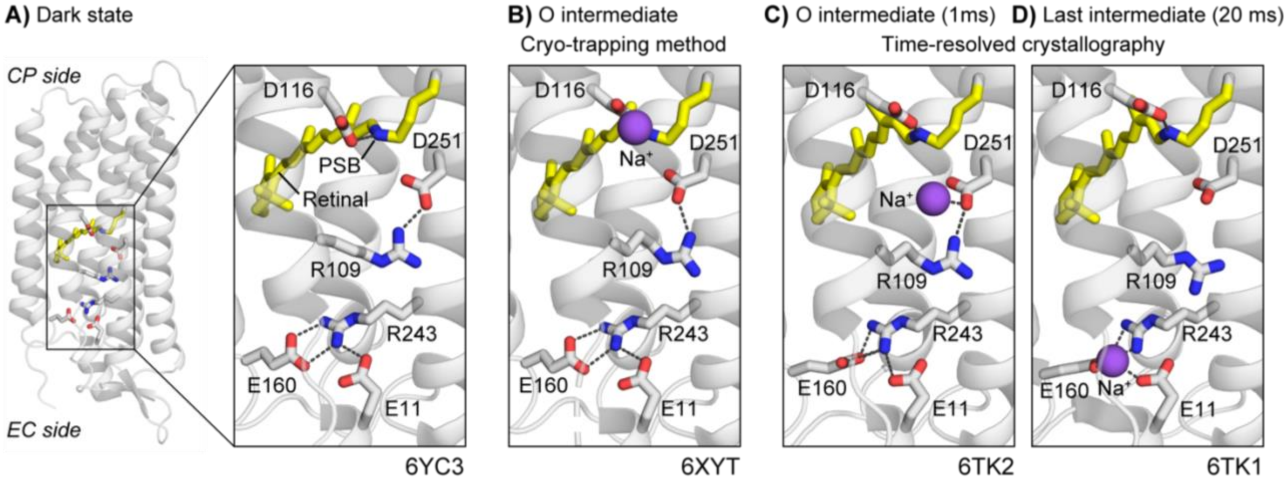

Figure S1. Tertiary structures of KR2. (A) Dark state, (B) O intermediate determined by the cryo-trapping method (11), (C) O intermediate and (D) the last intermediate determined by time-resolved X-ray crystallography (12). The latter two are the 1 ms and 20 ms structures after photoactivation. The respective PDB codes are indicated in the bottom right. Each enlargement shows two Asp residues (Asp116 and Asp251) that comprise respective Na^+ binding sites, the underlying Arg109 residue, and the complex of three residues (Glu11, Glu160, Arg243) at the EC surface. KR2, *Krokinobacter eikastus* rhodopsin 2; CP, cytoplasmic; EC, extracellular; PSB, protonated Schiff base.

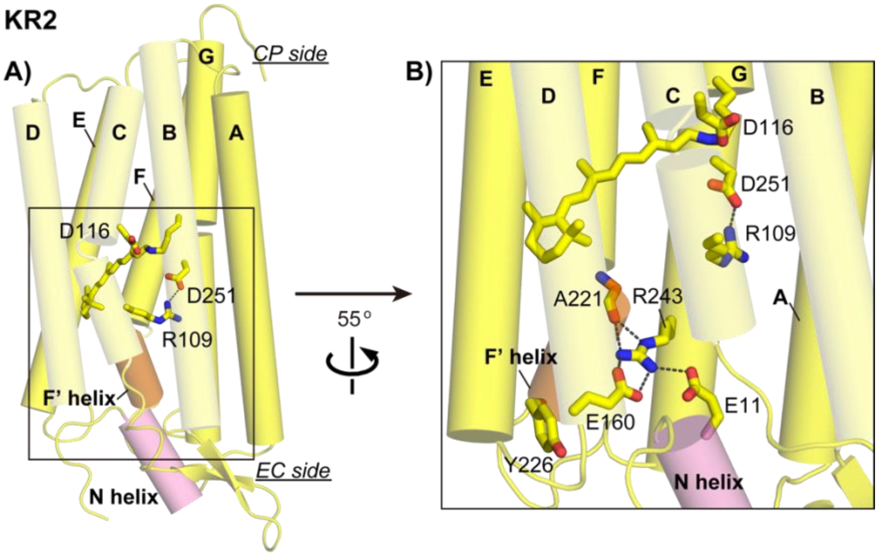

Figure S2. KR2 structure in the dark state. The PDB code is 6YC3. The overall structure is shown in (A), and its boxed region is rotated 55° and enlarged in (B). The corresponding figures for the IaNaR protomers are shown in Fig. 3. The Arg109 residue and the three-residue complex (Glu11, Glu160, Arg243) take essentially the same arrangements as IaNaR molecule A. KR2, *Krokinobacter eikastus* rhodopsin 2; IaNaR, *Inidibacter alkaliphilus* sodium pump rhodopsin; CP, cytoplasmic; EC, extracellular.

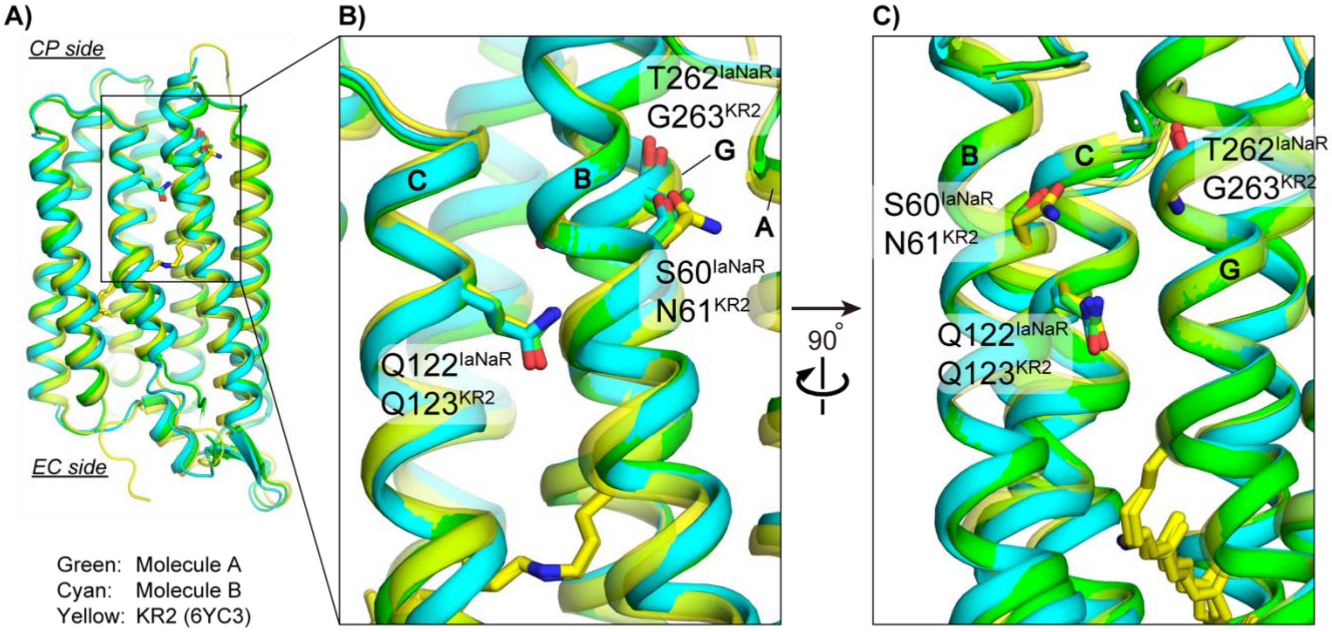

Figure S3. Comparison of CP side structures among two IaNaR protomers and KR2. (A) Overall structures viewed parallel to the membrane. The boxed areas are enlarged in (B) and (C) with different angles. IaNaR protomers are colored green (molecule A) and cyan (molecule B), and KR2 is colored yellow. On the CP sides, there are no essential differences in the positions of Cα atoms among the three molecules. Three important residues, comprising the Na^+ transport pathway, are shown in the stick model (8,17). Their positions are also superimposed. The A helices are not shown in (C). IaNaR, *Inidibacter alkaliphilus* sodium pump rhodopsin; KR2, *Krokinobacter eikastus* rhodopsin 2; CP, cytoplasmic; EC, extracellular.

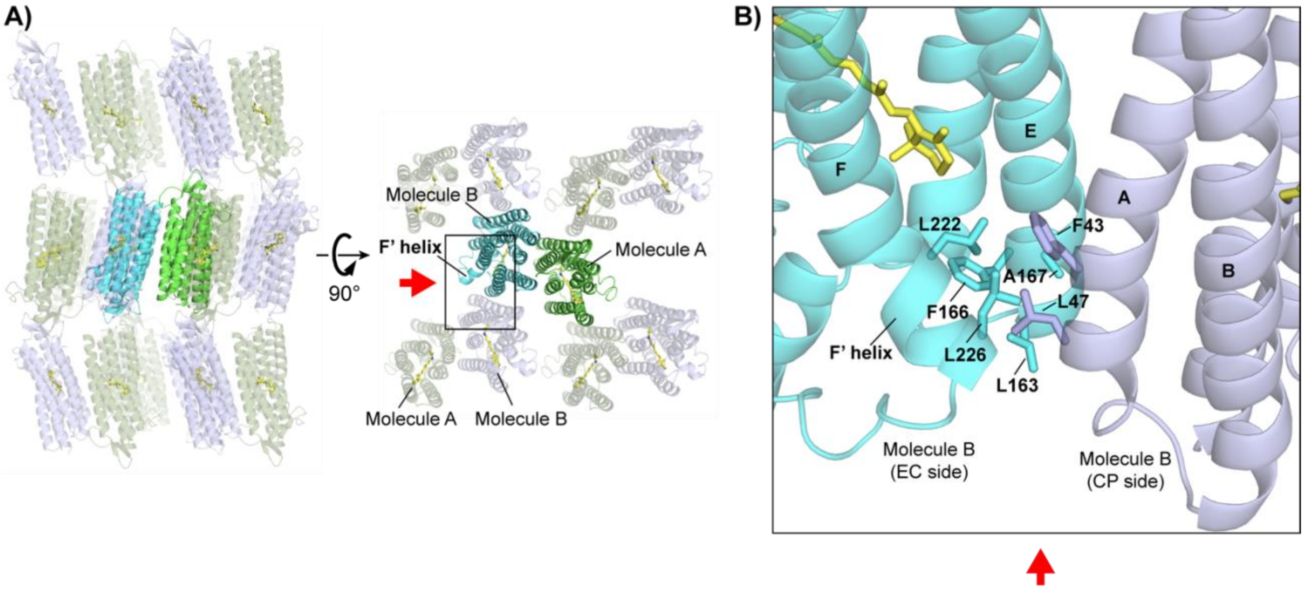

Figure S4. Inter protomer interaction of IaNaR. (A) Crystal packing of IaNaR viewed parallel to the membrane (left) and normal to the membrane surface (right). The asymmetric unit consists of an antiparallel dimer. Molecules A and B in the central unit are colored green and cyan, and those in other units are pale green and pale purple. (B) The boxed region in (A) is enlarged with a different angle. The F' helix in molecule B (cyan) forms a hydrophobic interaction with the CP end of the A helix from molecule B (purple) involved in the neighboring asymmetric unit. IaNaR, *Inidibacter alkaliphilus* sodium pump rhodopsin.

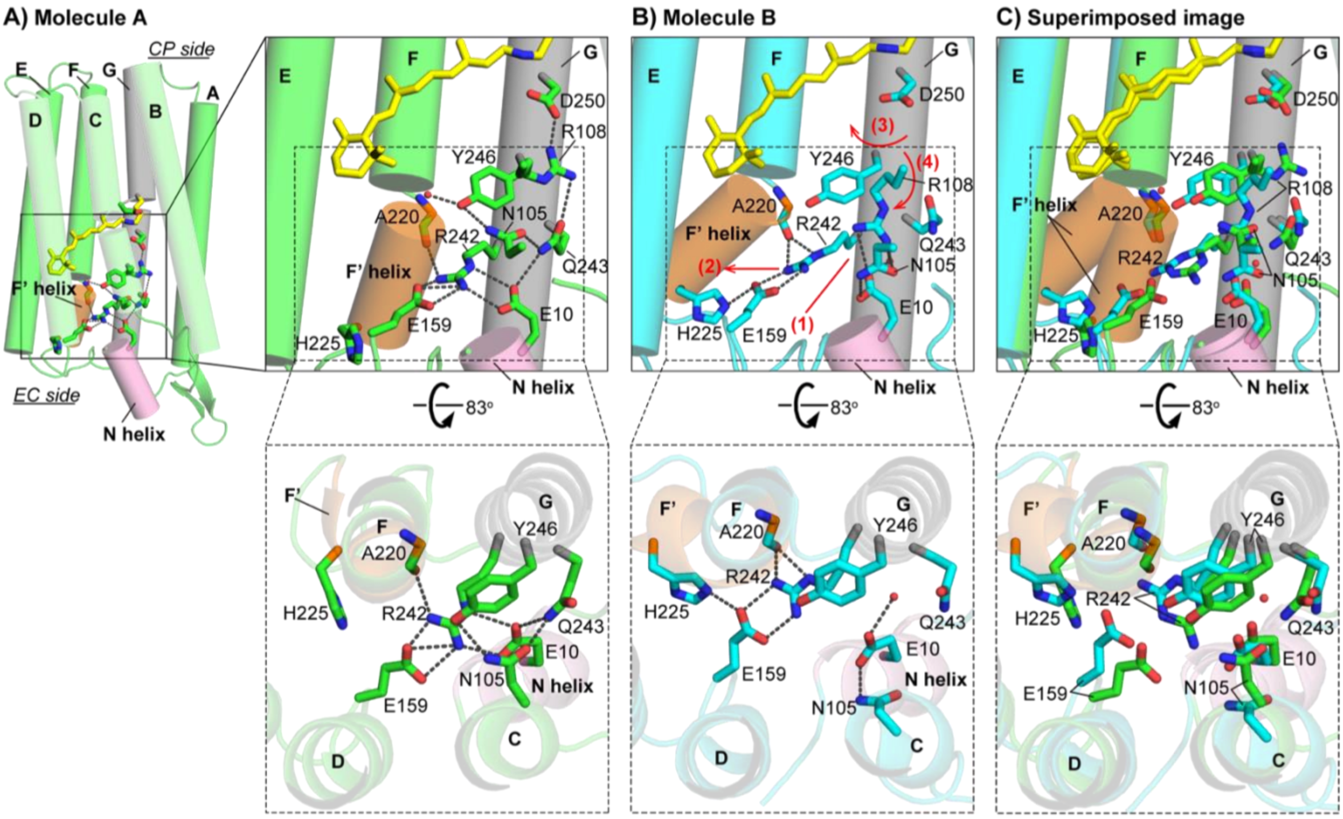

Figure S5. Detailed comparison between the EC half structures of molecules A and B. The same panels in Fig. 5 are shown in (A) and (B), along with their superimposed images in (C). CP, cytoplasmic; EC, extracellular.

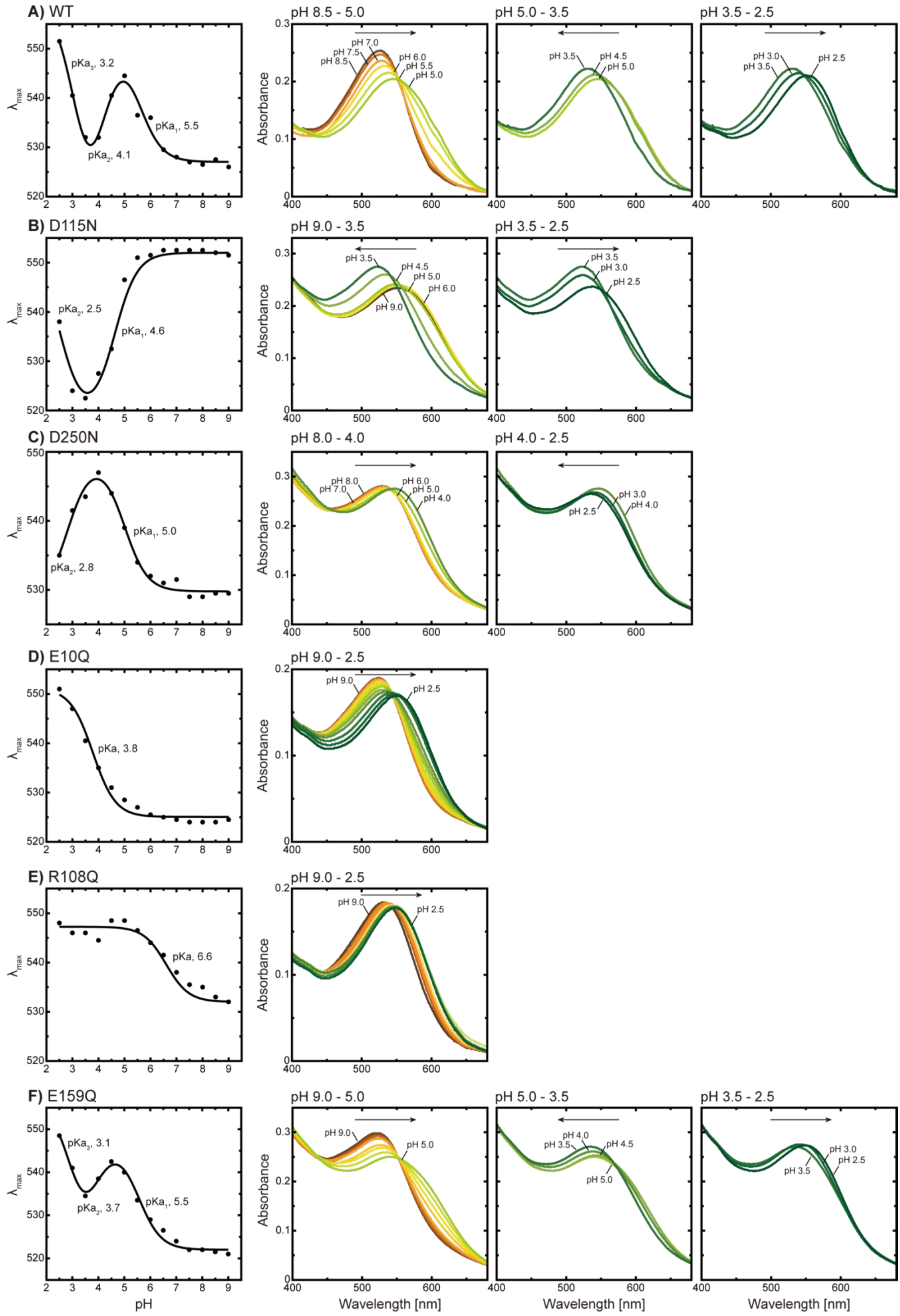
A) WT
pKa3, 3.2
pKa2, 4.1
pKa1, 5.5
pH 8.5 - 5.0
pH 8.5
pH 7.5
pH 7.0
pH 6.0
pH 5.5
pH 5.0
pH 5.0 - 3.5
pH 3.5
pH 4.5
pH 5.0
pH 3.5 - 2.5
pH 3.0
pH 3.5
pH 2.5
B) D115N
pKa2, 2.5
pKa1, 4.6
pH 9.0 - 3.5
pH 3.5
pH 4.5
pH 5.0
pH 6.0
pH 9.0
pH 3.5 - 2.5
pH 3.5
pH 3.0
pH 2.5
C) D250N
pKa1, 5.0
pKa2, 2.8
pH 8.0 - 4.0
pH 8.0
pH 7.0
pH 6.0
pH 5.0
pH 4.0
pH 4.0 - 2.5
pH 3.0
pH 4.0
pH 2.5
D) E10Q
pKa, 3.8
pH 9.0 - 2.5
pH 9.0
pH 2.5
E) R108Q
pKa, 6.6
pH 9.0 - 2.5
pH 9.0
pH 2.5
F) E159Q
pKa3, 3.1
pKa2, 3.7
pKa1, 5.5
pH 9.0 - 5.0
pH 9.0
pH 5.0
pH 5.0 - 3.5
pH 4.0
pH 3.5
pH 4.5
pH 5.0
pH 3.5 - 2.5
pH 3.0
pH 2.5
pH 3.5
λmax
Absorbance
pH
Wavelength [nm]

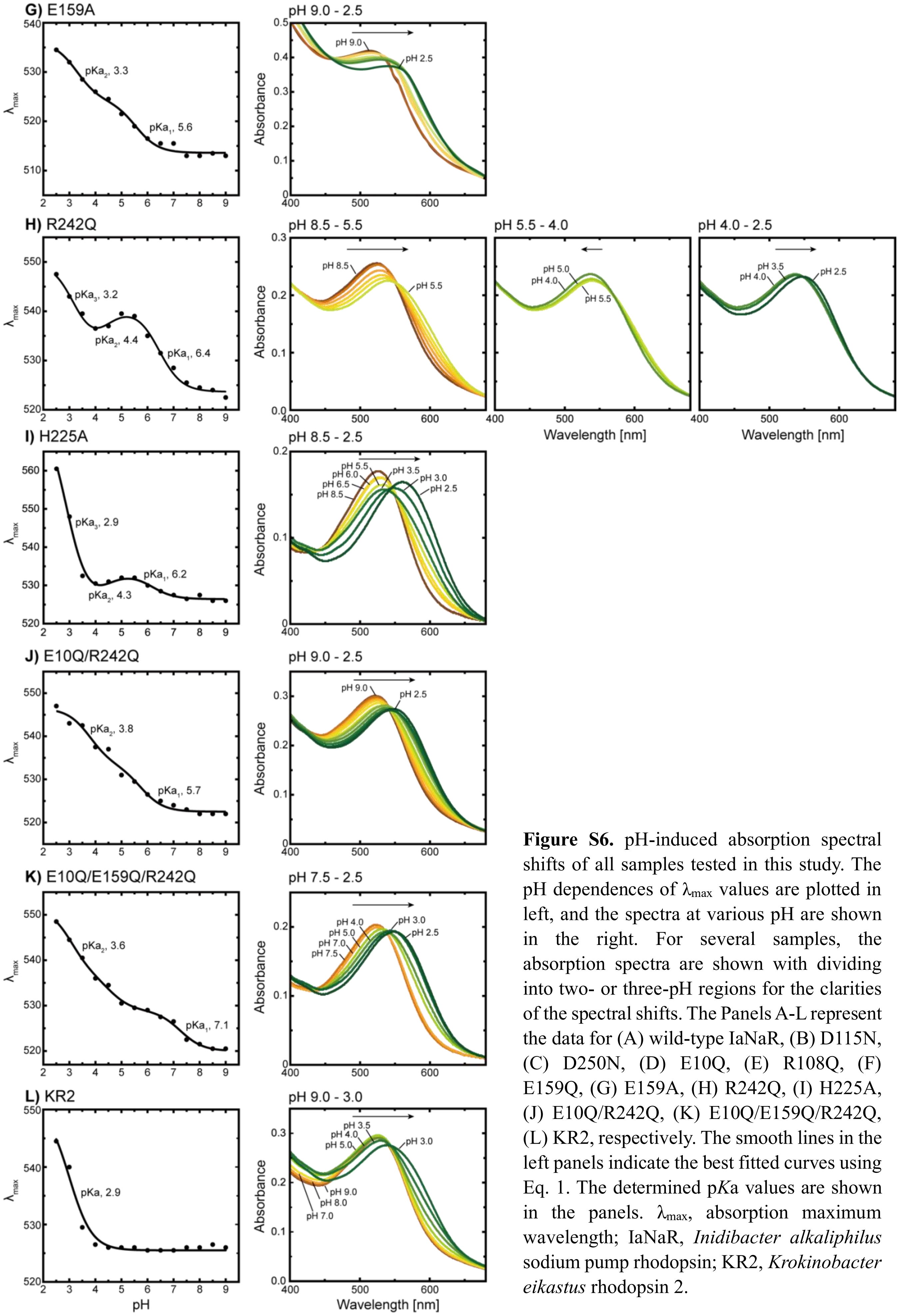

Figure S6. pH-induced absorption spectral shifts of all samples tested in this study. The pH dependences of λ_{max} values are plotted in left, and the spectra at various pH are shown in the right. For several samples, the absorption spectra are shown with dividing into two- or three-pH regions for the clarities of the spectral shifts. The Panels A-L represent the data for (A) wild-type IaNaR, (B) D115N, (C) D250N, (D) E10Q, (E) R108Q, (F) E159Q, (G) E159A, (H) R242Q, (I) H225A, (J) E10Q/R242Q, (K) E10Q/E159Q/R242Q, (L) KR2, respectively. The smooth lines in the left panels indicate the best fitted curves using Eq. 1. The determined p*K*a values are shown in the panels. λ_{max}, absorption maximum wavelength; IaNaR, *Inidibacter alkaliphilus* sodium pump rhodopsin; KR2, *Krokinobacter eikastus* rhodopsin 2.

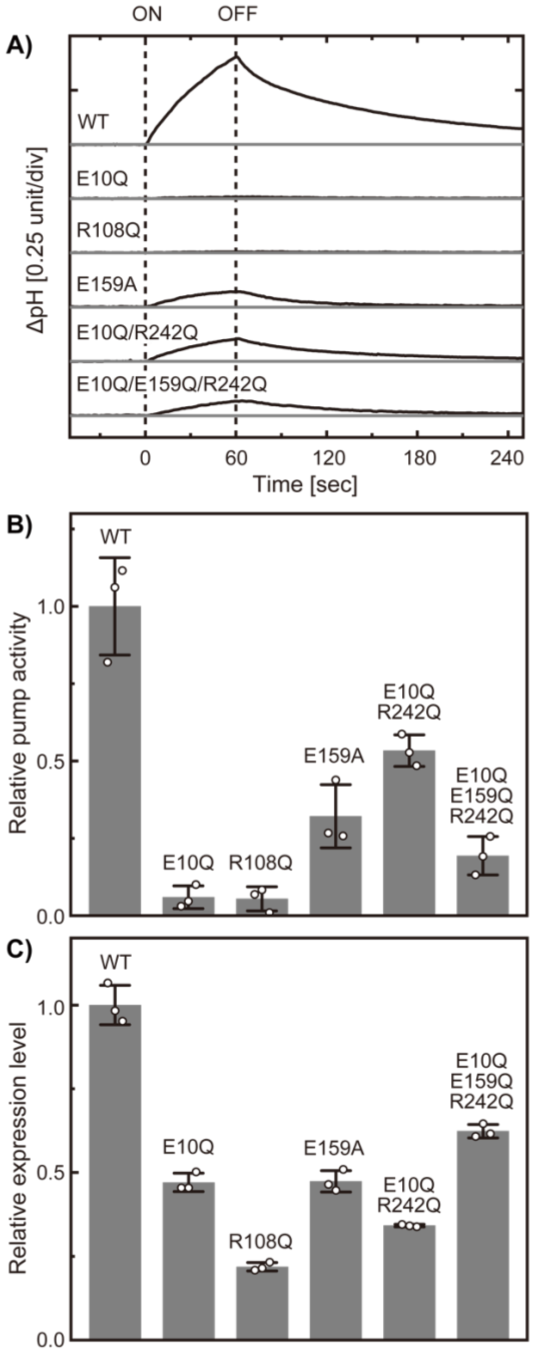

Figure S7. Na^+ pumping activities of all samples tested in this study. The data shown in Figs. 6 and 10 are summarized here supplemented with several time courses of pH changes. (A) Time course of light-induced pH changes in *E. coli* suspensions. The light is turned on and off at 0 and 60 s, respectively. (B) The relative pump activities calculated by dividing the initial slopes of light-induced pH changes by the respective expression levels. (C) The relative expression levels in the *E. coli* cell membranes determined by the flash-induced absorbance changes at λ_{max} in the dark state. The *E. coli* cells after the measurements of pH changes were used for the samples. In (B) and (C), the scatter of open circles represents each measurement, and the bar represents the mean ± standard deviation (n = 3). λ_{max}, absorption maximum wavelength.

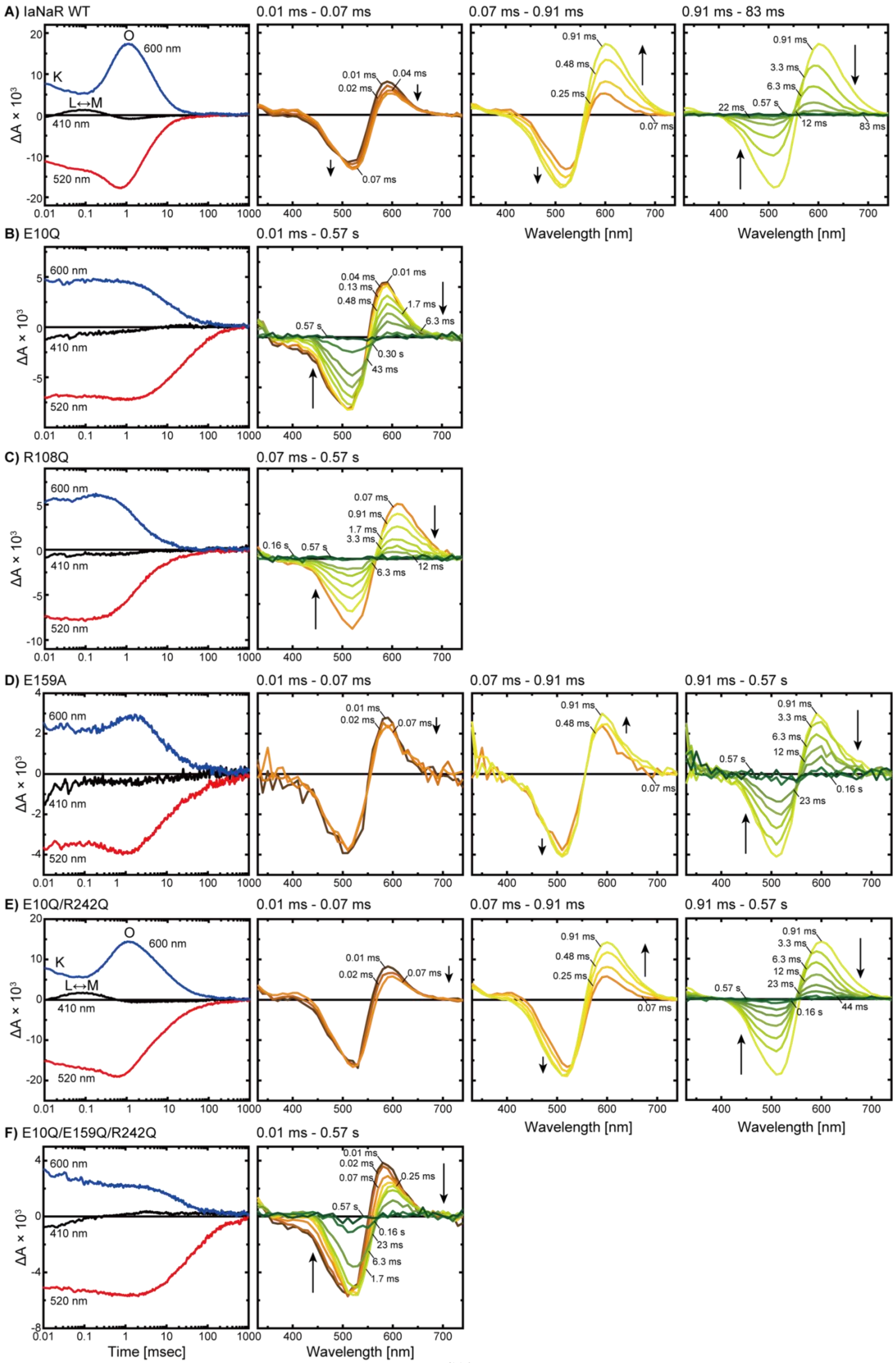
A) IaNaR WT
0.01 ms - 0.07 ms
0.07 ms - 0.91 ms
0.91 ms - 83 ms
O
600 nm
K
L↔M
410 nm
520 nm
ΔA × 10^3
Wavelength [nm]
B) E10Q
0.01 ms - 0.57 s
C) R108Q
0.07 ms - 0.57 s
D) E159A
0.01 ms - 0.07 ms
0.07 ms - 0.91 ms
0.91 ms - 0.57 s
E) E10Q/R242Q
0.01 ms - 0.07 ms
0.07 ms - 0.91 ms
0.91 ms - 0.57 s
F) E10Q/E159Q/R242Q
0.01 ms - 0.57 s
Time [msec]
Wavelength [nm]

Figure S8. Flash-induced absorbance changes of all samples tested in this study. The time courses of absorbance changes at three typical wavelengths are shown on the left, and the light minus dark difference spectra are shown on the right. For several samples, the difference spectra are divided into two- or three-time domains to clarify the spectral changes. Panels A-F represent the data for (A) wild-type IaNaR, (B) E10Q, (C) R108Q, (D) E159A, (E) E10Q/R242Q, and (F) E10Q/E159Q/R242Q, respectively. IaNaR, *Inidibacter alkaliphilus* sodium pump rhodopsin.

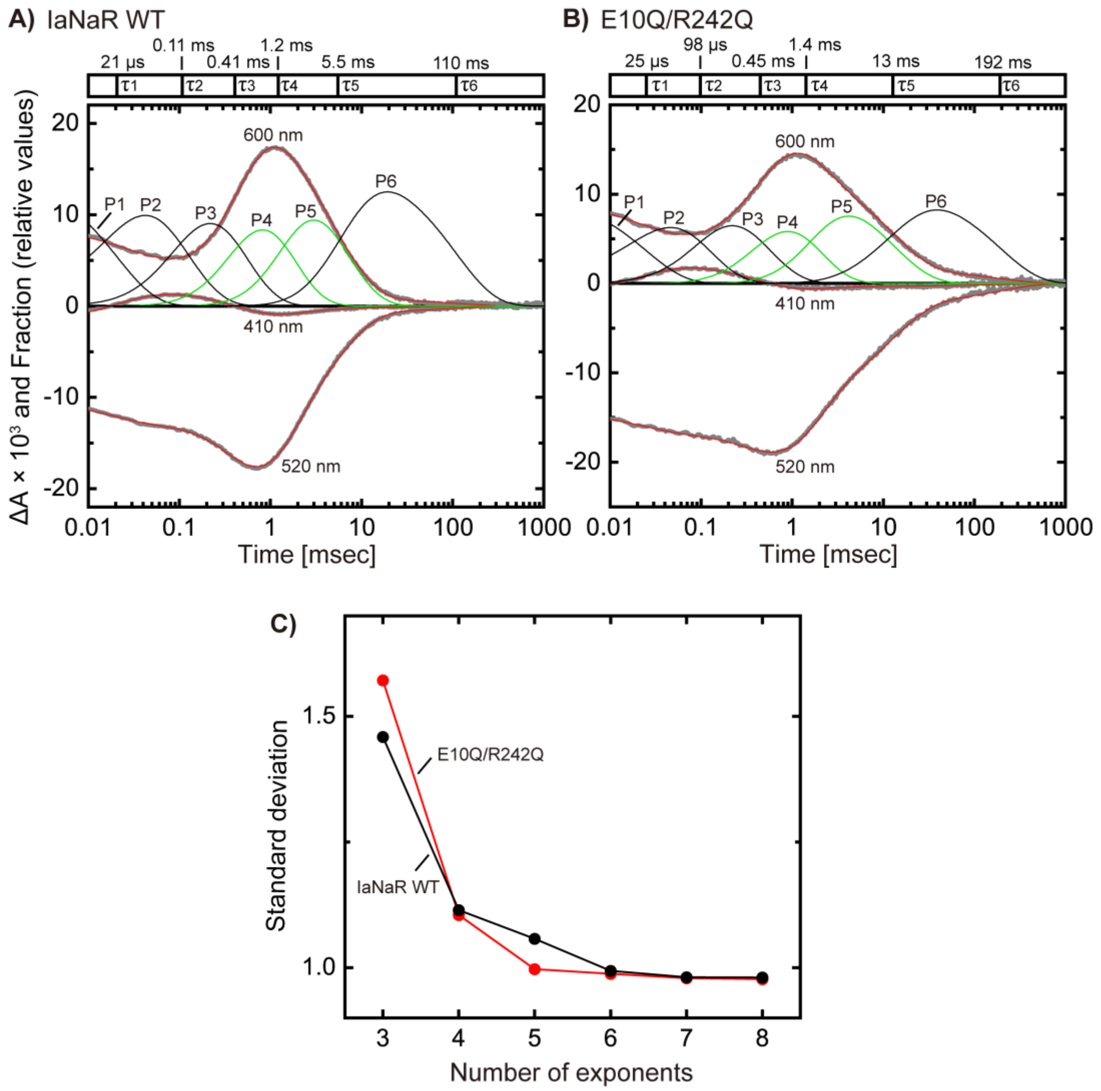

Figure S9. Analysis results of the flash-induced absorbance changes. Panels (A) and (B) represent the fitting results with 6 exponents for wild-type IaNaR and the E10Q/R242Q mutant, respectively. The top thin panels indicate the decay time constants of P*i* states (*i*=1-6). The large panels contain the raw data (grey lines), fitting curves (red lines) and the calculated concentration changes of the P*i* states (black and green lines), respectively. Panel (C) indicates the standard deviations of the fitting residuals at various numbers of exponents. For wild-type IaNaR, the reduction of the standard deviation was saturated at 6 exponents. Thus, 6 exponents are necessary to describe the photocycle. In contrast, the corresponding reduction for the E10Q/R242Q mutant was saturated at 5 exponents. Thus, 5 exponents are enough for this mutant. However, in both panels (A) and (B), 6 exponents were employed to facilitate comparison of their decay time constants. For the mutant, increasing the number of exponents from 5 to 6 split the P2 state into two states, which correspond to the P2 and P3 states in Panel (B). The other time constants were not significantly affected by the increase of the exponents.
